# Supplementary material for: Potent Chlorambucil-Platinum(IV) Prodrugs
Source: Int J Mol Sci. 2022 Sep 9;23(18):10471. doi: 10.3390/ijms231810471 (PMC9499463; doi:10.3390/ijms231810471)
Supplement: Supplementary file 1 [file ijms-23-10471-s001.zip › ijms-1858989-supplementary.pdf]

Supplementary Materials

Table of Contents

NMR spectra of precursor platinum(II) complexes

**Figure S1**	^{1}H-NMR, 2D-COSY and ^{1}H-^{195}Pt-HMQC spectra of **PHEN*SS*** in D_2O obtained at 298 K. Inset: structure of **PHEN*SS*** with proton labeling system.	**6**
**Figure S2**	^{1}H-NMR, 2D-COSY and ^{1}H-^{195}Pt-HMQC spectra of **5ME*SS*** in D_2O obtained at 298 K. Inset: structure of **5ME*SS*** with proton labeling system.	**7**
**Figure S3**	^{1}H-NMR, 2D-COSY and ^{1}H-^{195}Pt-HMQC spectra of **56ME*SS*** in D_2O obtained at 298 K. Inset: structure of **56ME*SS*** with proton labeling system.	**7**

NMR spectra of precursor platinum(IV) complexes

**Figure S4**	^{1}H-NMR, 2D-COSY and ^{1}H-^{195}Pt-HMQC spectra of **PHEN*SS*(IV)(OH)$_2$** in D_2O obtained at 298 K. Inset: structure of **PHEN*SS*(IV)(OH)$_2$** with proton labeling system.	**8**
**Figure S5**	^{1}H-NMR, 2D-COSY and ^{1}H-^{195}Pt-HMQC spectra of **5ME*SS*(IV)(OH)$_2$** in D_2O obtained at 298 K. Inset: structure of **5ME*SS*(IV)(OH)$_2$** with proton labeling system.	**9**
**Figure S6**	^{1}H-NMR, 2D-COSY and ^{1}H-^{195}Pt-HMQC spectra of **56ME*SS*(IV)(OH)$_2$** in D_2O obtained at 298 K. Inset: structure of **56ME*SS*(IV)(OH)$_2$** with proton labeling system.	**9**

HPLC chromatograms

**Figure S7**	HPLC chromatogram of CLB anhydride (at 17 min) with DCU by-product (at 15.9 min), within the collection wavelength of 254 nm obtained at 298 K, by an Agilent ZORBAX RX-C_{18} column (100 × 4.6 mm, 3.5 μm pore size).	**10**
**Figure S8**	HPLC chromatogram of **PCLB** within the collection wavelength of 254 nm obtained at 298 K, by a Phenomenex Onyx™ Monolithic C_{18}-reverse phase column (100 × 4.6 mm, 5 μm pore size).	**10**

**Figure S9**	HPLC chromatogram of **5CLB** within the collection wavelength of 254 nm obtained at 298 K, by a Phenomenex Onyx™ Monolithic C_{18}-reverse phase column (100 × 4.6 mm, 5 μm pore size).	**11**
**Figure S10**	HPLC chromatogram of **56CLB** within the collection wavelength of 254 nm obtained at 298 K, by a Phenomenex Onyx™ Monolithic C_{18}-reverse phase column (100 × 4.6 mm, 5 μm pore size).	**11**

NMR spectra of platinum(IV)-CLB complexes

**Figure S11**	^{1}H-NMR spectrum of **PCLB** in D_2O obtained at 298 K. Inset: structure of **PCLB** with proton labeling system.	**12**
**Figure S12**	^{1}H-NMR spectrum of **5CLB** in D_2O obtained at 298 K. Inset: structure of **5CLB** with proton labeling system.	**13**
**Figure S13**	^{1}H-NMR spectrum of **56CLB** in D_2O obtained at 298 K. Inset: structure of **56CLB** with proton labeling system.	**13**
**Figure S14**	2D-COSY spectrum of **PCLB** in D_2O obtained at 298 K. Inset: structure of **PCLB** with proton labeling system.	**14**
**Figure S15**	2D-COSY spectrum of **5CLB** in D_2O obtained at 298 K. Inset: structure of **5CLB** with proton labeling system.	**14**
**Figure S16**	2D-COSY spectrum of **56CLB** in D_2O obtained at 298 K. Inset: structure of **56CLB** with proton labeling system.	**15**
**Figure S17**	^{1}H-^{195}Pt-HMQC spectrum of **PCLB** in D_2O obtained at 298 K. Region: 400 ppm. Inset: structure of **PCLB** with proton labelling system and arrows that indicate coupling.	**16**
**Figure S18**	^{1}H-^{195}Pt-HMQC spectrum of **PCLB** in D_2O obtained at 298 K. Region: -2800 ppm. Absence of platinum(II) resonance.	**17**
**Figure S19**	^{1}H-^{195}Pt-HMQC spectrum of **5CLB** in D_2O obtained at 298 K. Region: 400 ppm. Inset: structure of **5CLB** with proton labelling system and arrow that indicate coupling.	**18**
**Figure S20**	^{1}H-^{195}Pt-HMQC spectrum of **5CLB** in D_2O obtained at 298 K. Region: -2800 ppm. Absence of platinum(II) resonance.	**19**
**Figure S21**	^{1}H-^{195}Pt-HMQC spectrum of **56CLB** in D_2O obtained at 298 K. Region: 400 ppm. Inset: structure of **56CLB** with proton labelling system and arrows that indicate coupling.	**20**

**Figure S22**	^{1}H-^{195}Pt-HMQC spectrum of **56CLB** in D_2O obtained at 298 K. Region: -2800 ppm. Absence of platinum(II) resonance.	**21**

UV spectra of platinum(IV)-CLB complexes

**Figure S23**	UV spectra of **PCLB** obtained at 298 K. Inset: structure of **PCLB** and generated plot curves within the wavelengths 203 and 279 nm at 298 K.	**22**
**Figure S24**	UV spectra of **5CLB** obtained at 298 K. Inset: structure of **5CLB** and generated plot curves within the wavelengths 204 and 284 nm at 298 K.	**23**
**Figure S25**	UV spectra of **56CLB** obtained at 298 K. Inset: structure of **56CLB** and generated plot curves within the wavelengths 204 and 291 nm at 298 K.	**23**

CD spectra of platinum(IV)-CLB complexes

**Figure S26**	CD spectrum of **PCLB** in d.i.H_2O obtained at 298 K. Inset: structure of **PCLB**.	**24**
**Figure S27**	CD spectrum of **5CLB** in d.i.H_2O obtained at 298 K. Inset: structure of **5CLB**.	**25**
**Figure S28**	CD spectrum of **56CLB** in d.i.H_2O obtained at 298 K. Inset: structure of **56CLB**.	**26**

ESI-MS spectra of platinum(IV)-CLB complexes

**Figure S29**	ESI-MS spectrum of **PCLB** in d.i.H_2O obtained at 298 K, using a Waters SYNAPT G2-Si quadruple time-of-flight (QTOF) HDMS. Inset: structure of **PCLB**.	**27**
**Figure S30**	ESI-MS spectrum of **5CLB** in d.i.H_2O obtained at 298 K, using a Waters SYNAPT G2-Si quadruple time-of-flight (QTOF) HDMS. Inset: structure of **5CLB**.	**28**
**Figure S31**	ESI-MS spectrum of **56CLB** in d.i.H_2O obtained at 298 K, using a Waters SYNAPT G2-Si quadruple time-of-flight (QTOF) HDMS. Inset: structure of **56CLB**.	**29**

Stability experiments

Figure S32 Combined HPLC chromatograms of **PCLB**, **5CLB** and **56CLB** in three solvent systems, obtained at 298 K. Inset: structures of **PCLB**, **5CLB** and **56CLB**. **30**

Figure S33 Combined HPLC chromatograms of **PCLB**, **5CLB** and **56CLB** in three solvent systems, obtained at 298 K at 36 h. **31**

Lipophilicity measurements

Figure S34 Generated plot curve of log k *versus* concentration of organic solvent, CH_3CN to determine the chromatographic lipophilicity index, log k_w of the platinum(IV)-CLB complexes. **32**

Reduction reaction experiments

Figure S35 ESI-MS spectra of the reduced **PCLB** prodrug, highlighting the formation of the corresponding platinum(II) congener, **PHEN*SS***. **33**

Figure S36 ^{1}H-NMR spectra of **5CLB** with PBS and AsA in D_2O at 310.15 K, in different time intervals, highlighting the movement of resonances from the phenanthroline protons and the aromatic protons of the CLB ligand. **T** represents time in min. **34**

Figure S37 ^{1}H-NMR spectra of **56CLB** with PBS and AsA in D_2O at 310.15 K, in different time intervals, highlighting the movement of resonances from the phenanthroline protons and the aromatic protons of the CLB ligand. **T** represents time in min. **34**

Figure S38 1D-^{195}Pt NMR spectra of **5CLB** with PBS and AsA in D_2O at 310.15 K, within the regions of 400 and -2800 ppm, highlighting the complete reduction of the complex after 1 h. Inset: structures of **5CLB** and its platinum(II) scaffold, **5ME*SS***. **35**

Figure S39 1D-^{195}Pt NMR spectra of **56CLB** with PBS and AsA in D_2O at 310.15 K, within the regions of 400 and -2800 ppm, highlighting the complete reduction of the complex after 1 h. Inset: structures of **56CLB** and its platinum(II) scaffold, **56ME*SS***. **36**

Figure S40 ESI-MS spectra of the reduced **5CLB** prodrug, highlighting the formation of the corresponding platinum(II) congener, **5ME*SS***. **37**

Figure S41 ESI-MS spectra of the reduced **56CLB** prodrug, highlighting the formation of the corresponding platinum(II) congener, **56ME*SS***. **38**

ROS experiments

Figure S42: ROS production upon treatment with **PCLB**, **5CLB**, **56CLB**, cisplatin, together with precursor platinum(II) and (IV) scaffolds in HT29 colon cancer cell line at 0, 0.25, 0.5, 1, 3, 6, 12, 24, 48 and 72 h. TBHP: t-butyl hydroperoxide, NAC: *N*-acetylcysteine. Data points denote mean ± SEM. n = 3 from three independent experiments where samples were run in triplicates. **39**

NMR spectra of precursor platinum(II) complexes

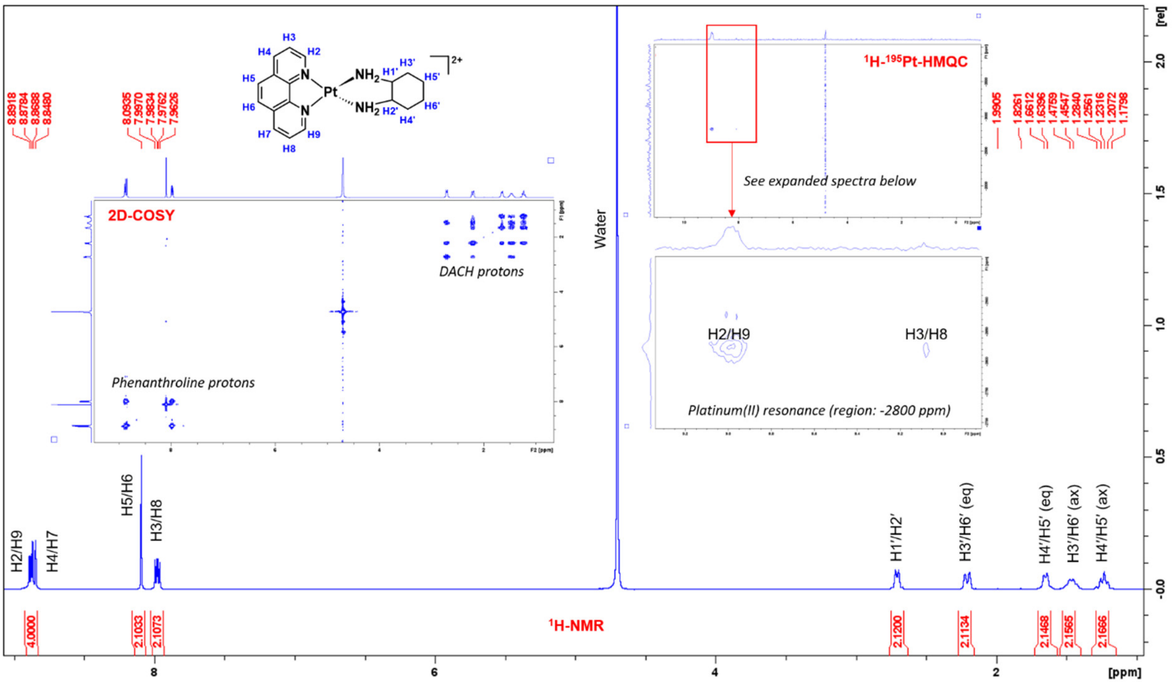

Figure S1: ^{1}H-NMR, 2D-COSY and ^{1}H-^{195}Pt-HMQC spectra of **PHEN*SS*** in D_2O obtained at 298 K. Inset: structure of **PHEN*SS*** with proton labeling system.

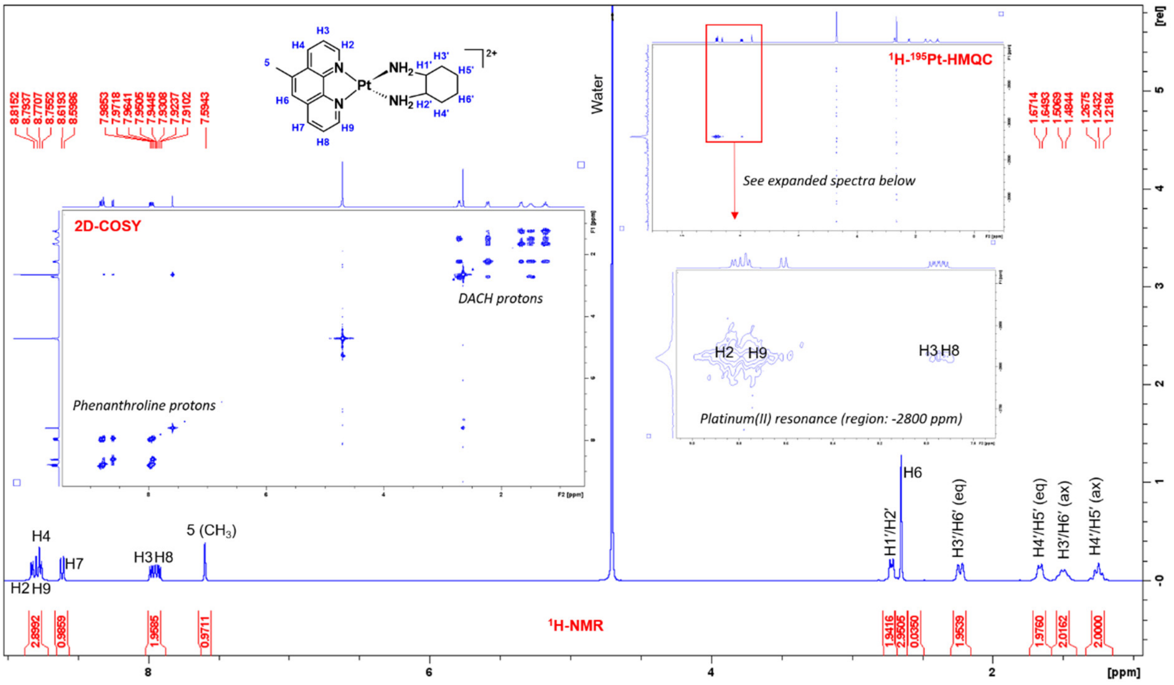

Figure S2: ^{1}H-NMR, 2D-COSY and ^{1}H-^{195}Pt-HMQC spectra of **5ME*SS*** in D_2O obtained at 298 K. Inset: structure of **5ME*SS*** with proton labeling system.

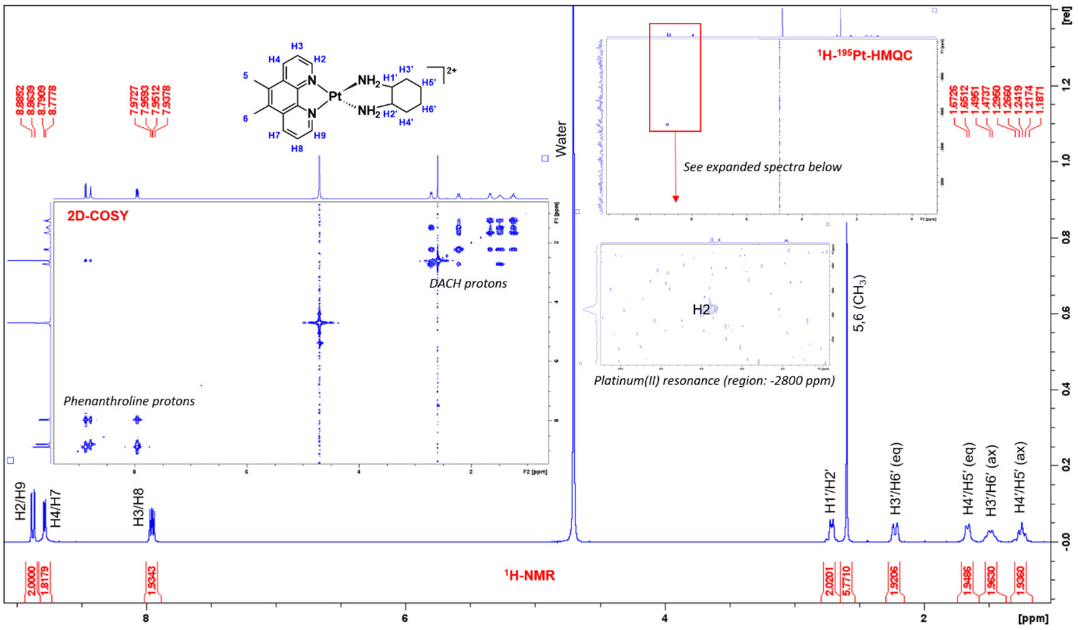

Figure S3: ^{1}H-NMR, 2D-COSY and ^{1}H-^{195}Pt-HMQC spectra of **56ME*SS*** in D_2O obtained at 298 K. Inset: structure of **56ME*SS*** with proton labeling system.

NMR spectra of precursor platinum(IV) dihydroxy complexes

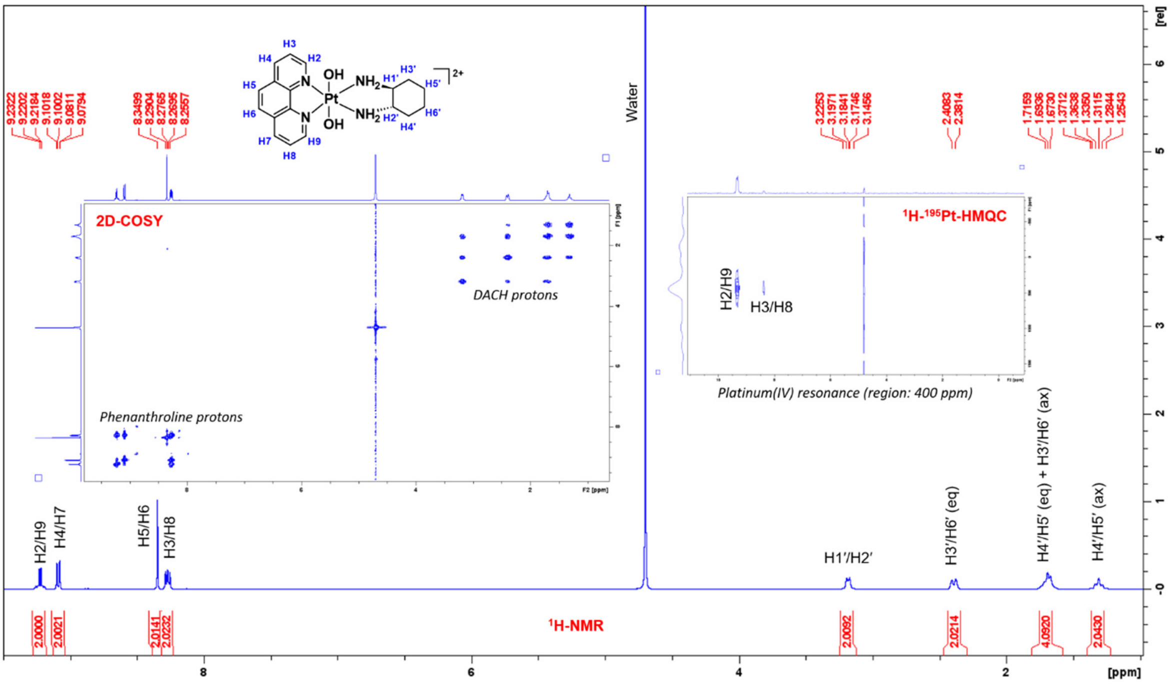

Figure S4: ^{1}H-NMR, 2D-COSY and ^{1}H-^{195}Pt-HMQC spectra of **PHEN*SS*(IV)(OH)$_2$** in D_2O obtained at 298 K. Inset: structure of **PHEN*SS*(IV)(OH)$_2$** with proton labeling system.

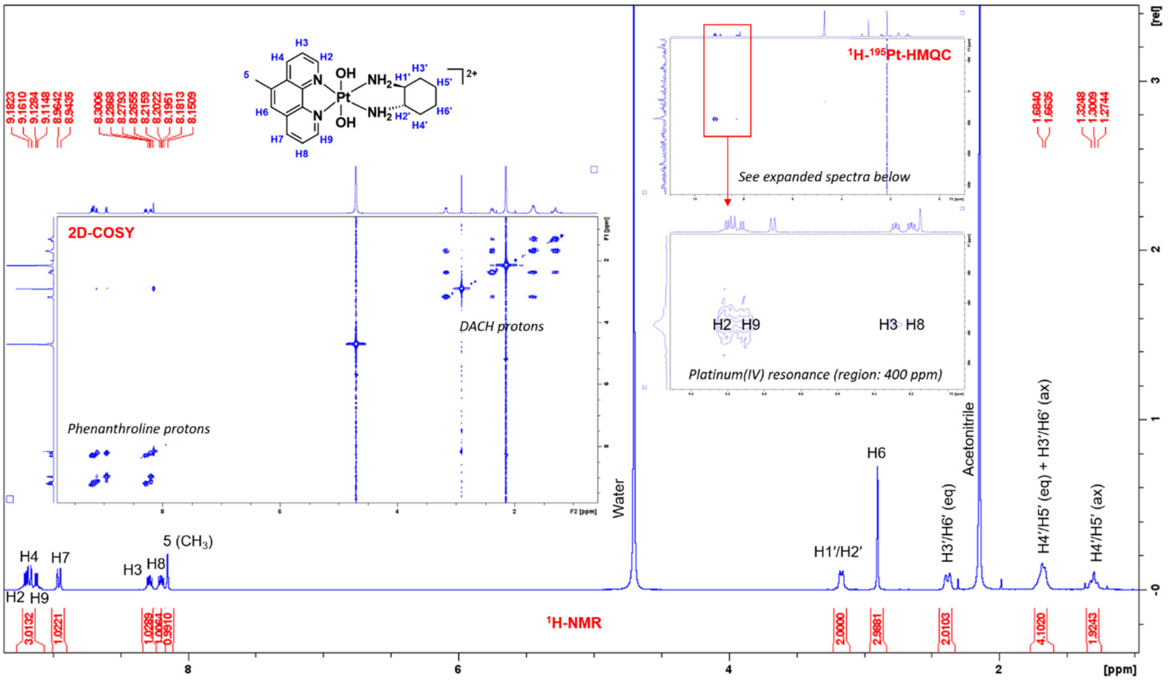

Figure S5: ^{1}H-NMR, 2D-COSY and ^{1}H-^{195}Pt-HMQC spectra of **5ME*SS*(IV)(OH)₂** in D_2O obtained at 298 K. Inset: structure of **5ME*SS*(IV)(OH)₂** with proton labeling system.

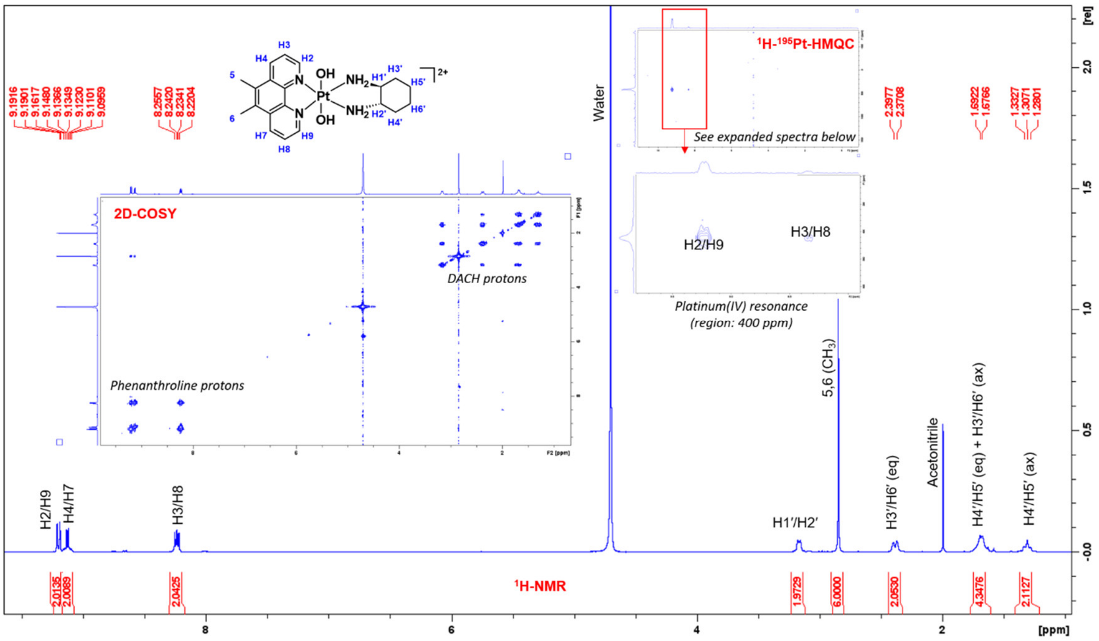

Figure S6: ^{1}H-NMR, 2D-COSY and ^{1}H-^{195}Pt-HMQC spectra of **56ME*SS*(IV)(OH)₂** in D_2O obtained at 298 K. Inset: structure of **56ME*SS*(IV)(OH)₂** with proton labeling system.

HPLC chromatogram of CLB anhydride

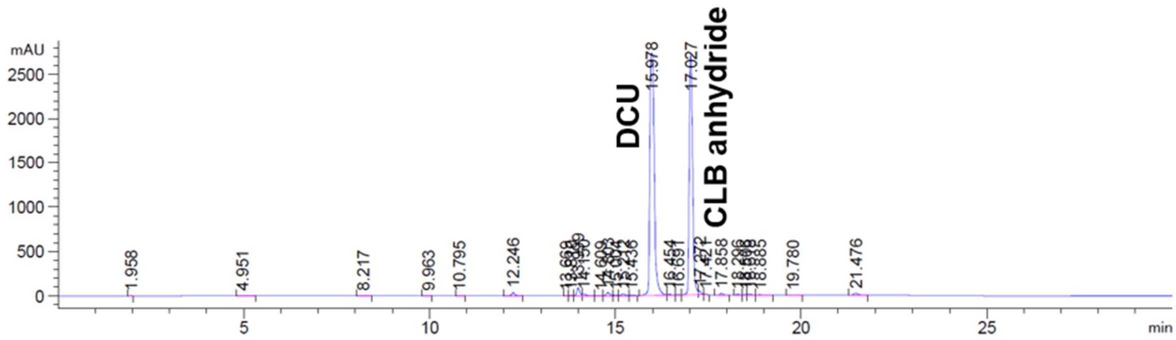

Figure S7: HPLC chromatogram of CLB anhydride (at 17 min) with DCU by-product (at 15.9 min), within the collection wavelength of 254 nm obtained at 298 K, by an Agilent ZORBAX RX-C_{18} column (100 × 4.6 mm, 3.5 μm pore size).

HPLC chromatograms of platinum(IV)-CLB complexes

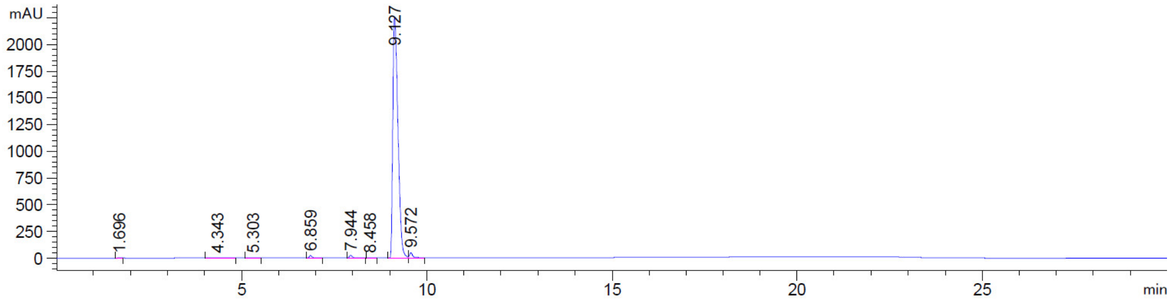

Figure S8: HPLC chromatogram of **PCLB** within the collection wavelength of 254 nm obtained at 298 K, by a Phenomenex Onyx™ Monolithic C_{18}-reverse phase column (100 × 4.6 mm, 5 μm pore size).

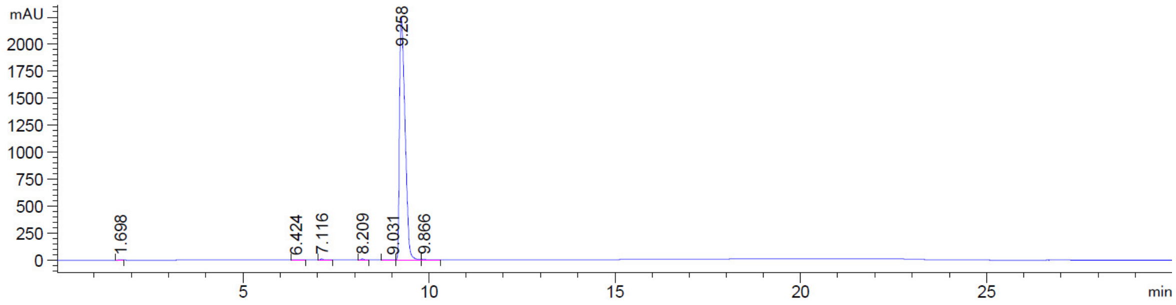

Figure S9: HPLC chromatogram of **5CLB** within the collection wavelength of 254 nm obtained at 298 K, by a Phenomenex Onyx™ Monolithic C_{18}-reverse phase column (100 × 4.6 mm, 5 µm pore size).

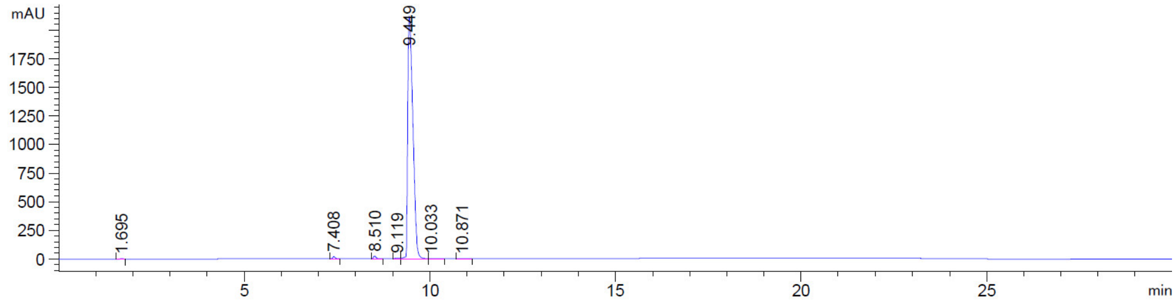

Figure S10: HPLC chromatogram of **56CLB** within the collection wavelength of 254 nm obtained at 298 K, by a Phenomenex Onyx™ Monolithic C_{18}-reverse phase column (100 × 4.6 mm, 5 µm pore size).

NMR spectra of platinum(IV)-CLB complexes

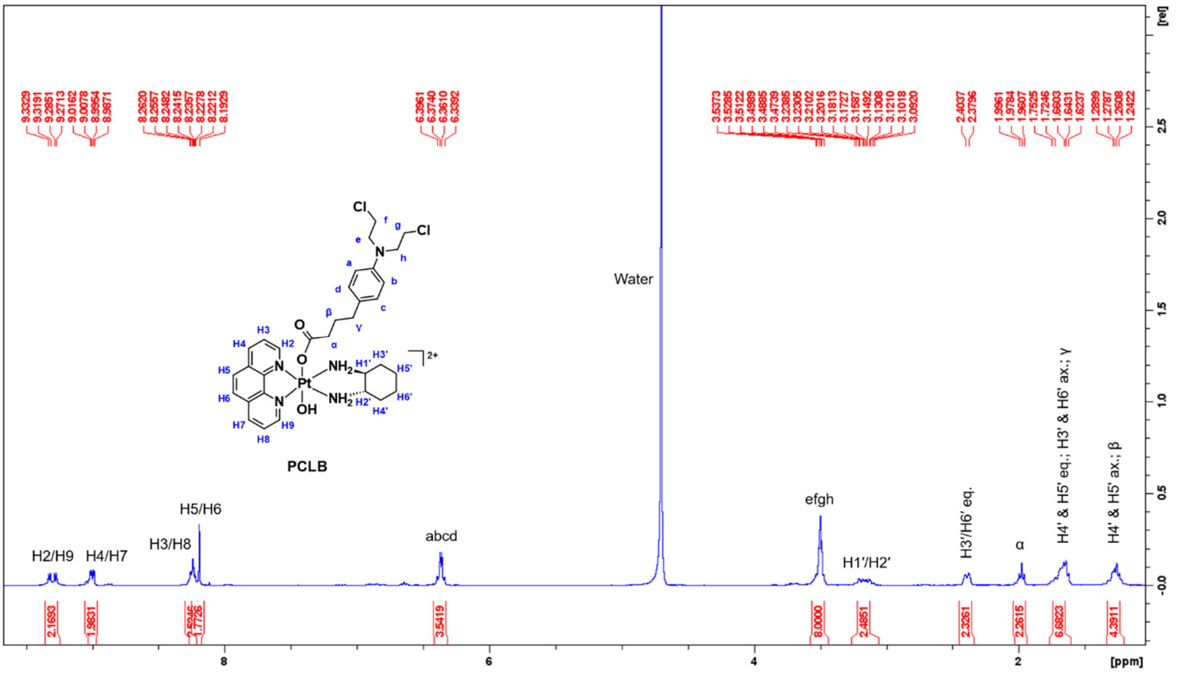

Figure S11: ^{1}H-NMR spectrum of **PCLB** in D_2O obtained at 298 K. Inset: structure of **PCLB** with proton labeling system.

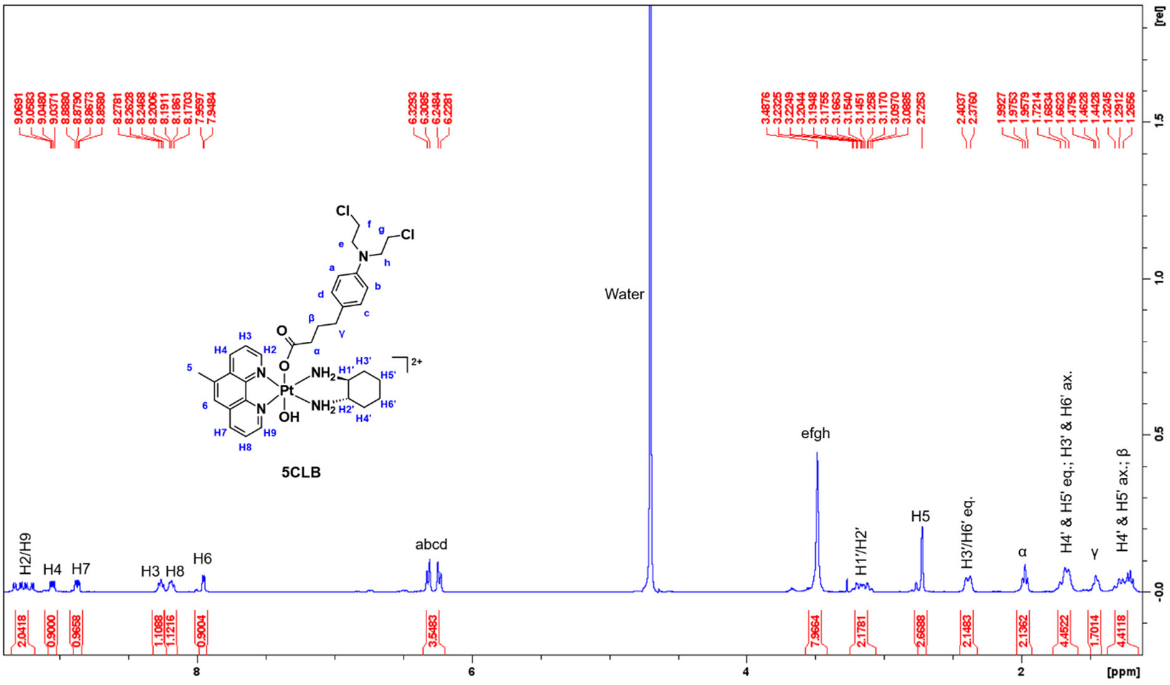

Figure S12: ^{1}H-NMR spectrum of **5CLB** in D_2O obtained at 298 K. Inset: structure of **5CLB** with proton labeling system.

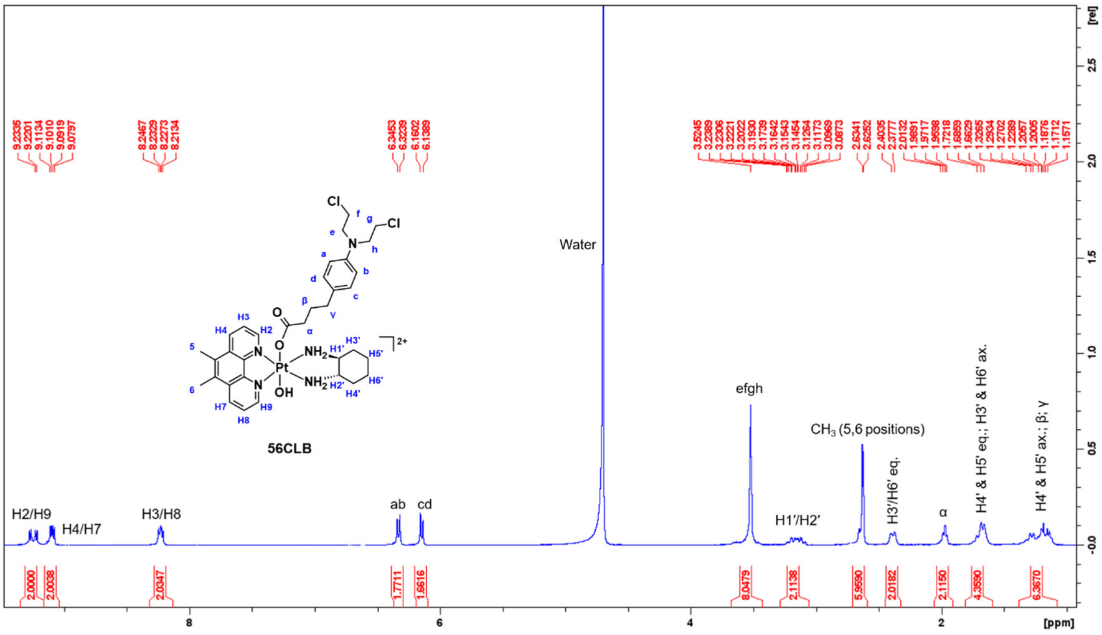

Figure S13: ^{1}H-NMR spectrum of **56CLB** in D_2O obtained at 298 K. Inset: structure of **56CLB** with proton labeling system.

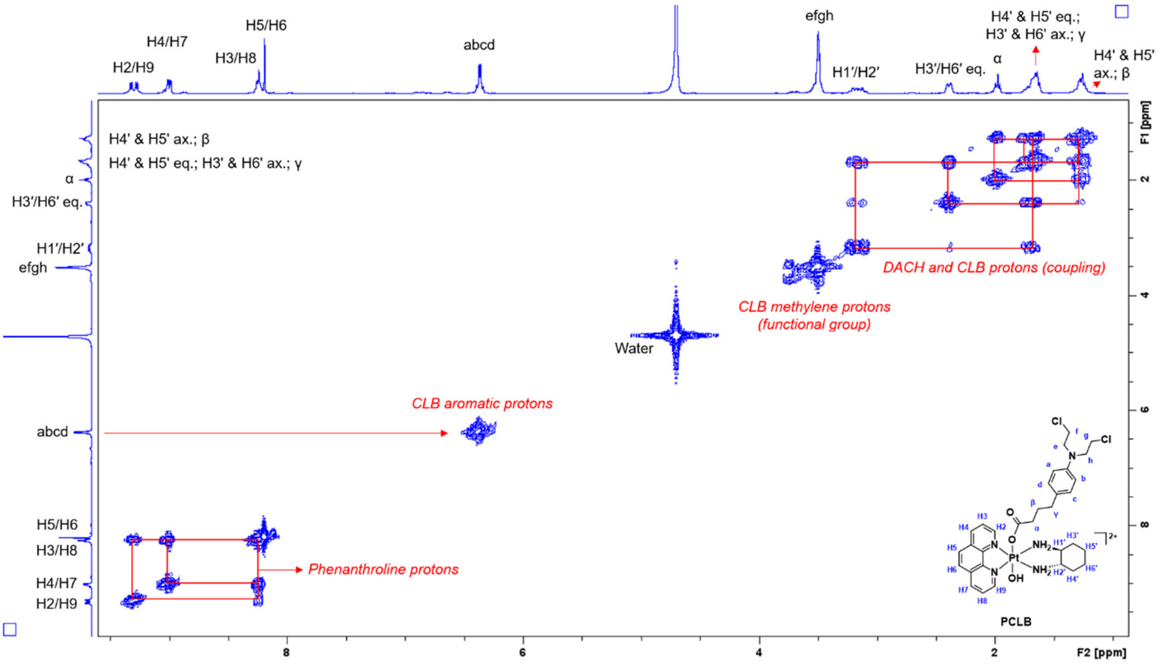

Figure S14: 2D-COSY spectrum of **PCLB** in D_2O obtained at 298 K. Inset: structure of **PCLB** with proton labeling system.

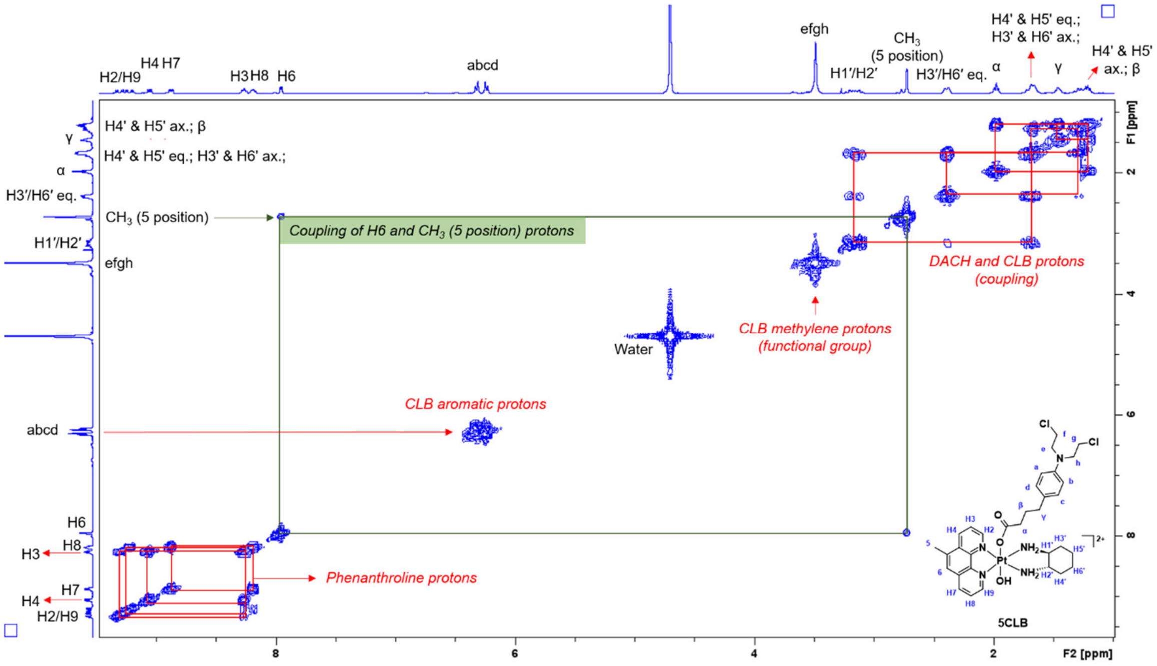

Figure S15: 2D-COSY spectrum of **5CLB** in D_2O obtained at 298 K. Inset: structure of **5CLB** with proton labeling system.

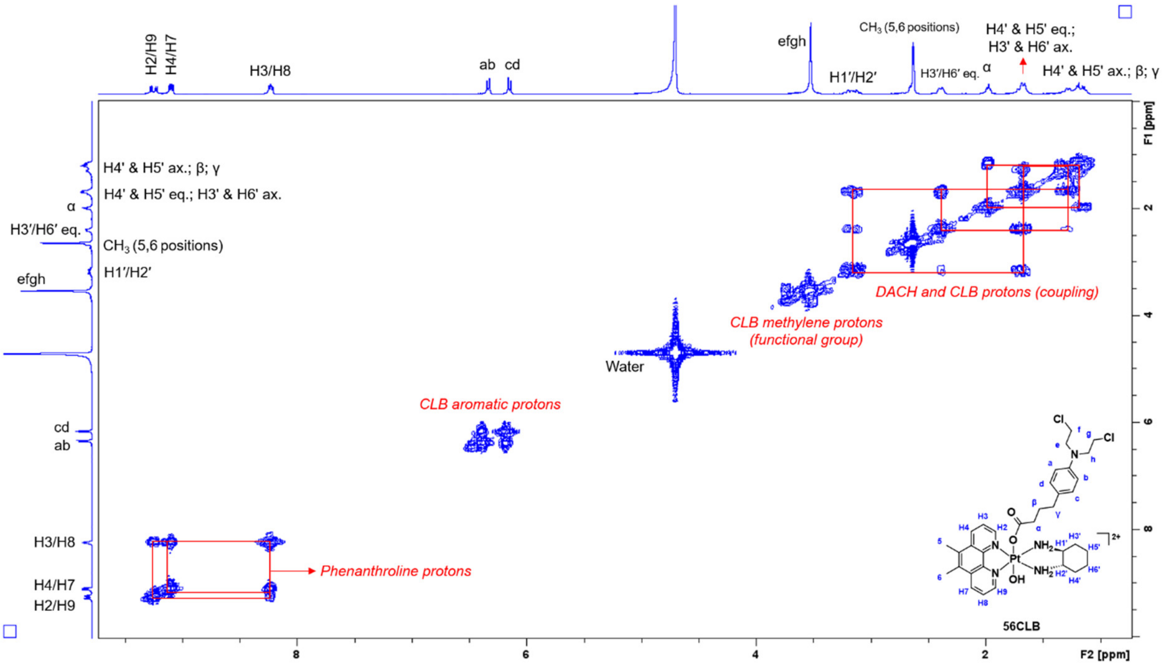

Figure S16: 2D-COSY spectrum of **56CLB** in D_2O obtained at 298 K. Inset: structure of **56CLB** with proton labeling system.

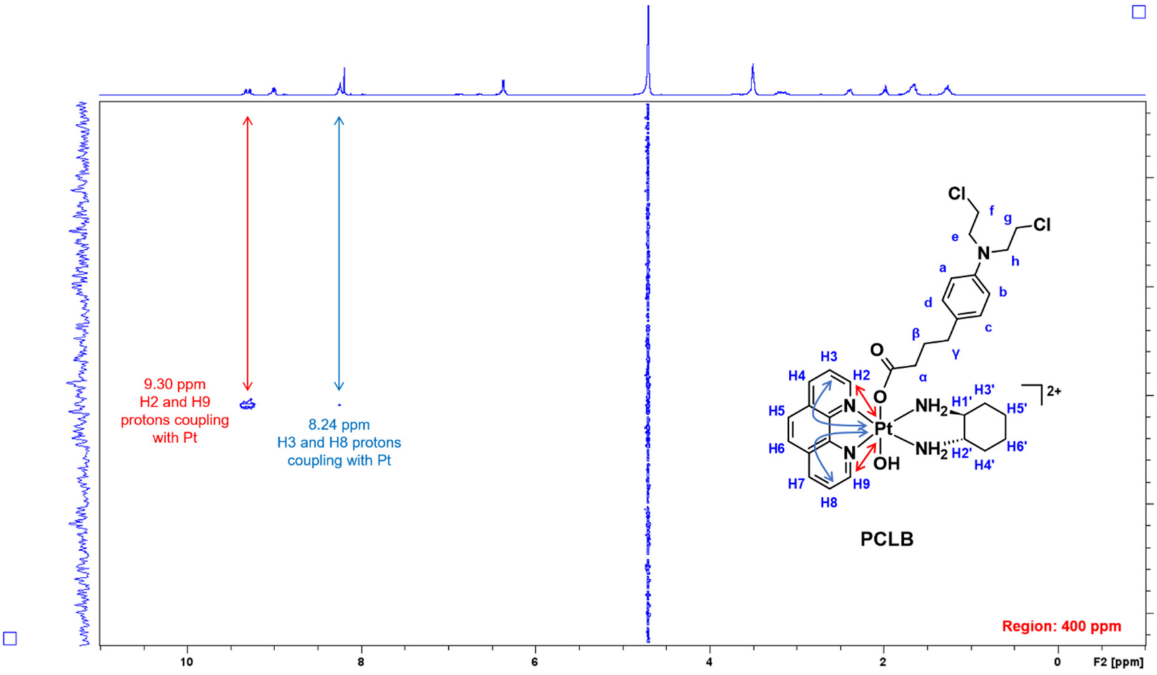

Figure S17: ^{1}H-^{195}Pt-HMQC spectrum of **PCLB** in D_2O obtained at 298 K. Region: 400 ppm. Inset: structure of **PCLB** with proton labelling system and arrows that indicate coupling.

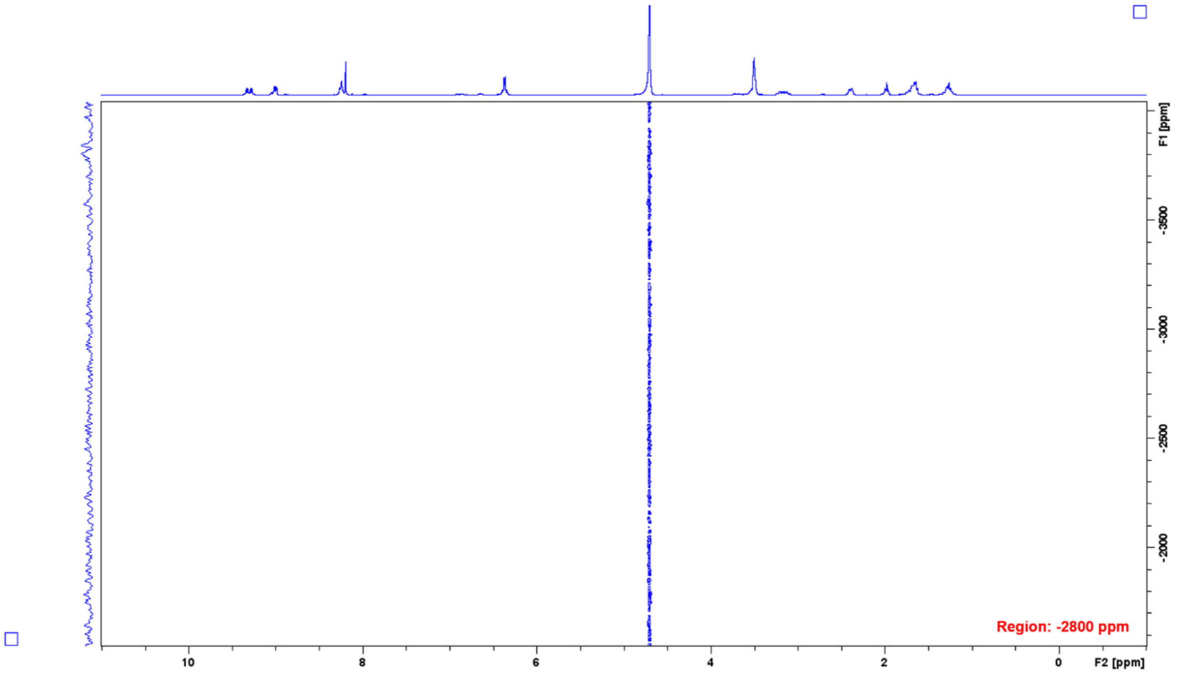

Figure S18: ^{1}H-^{195}Pt-HMQC spectrum of **PCLB** in D_2O obtained at 298 K. Region: -2800 ppm. Absence of platinum(II) resonance.

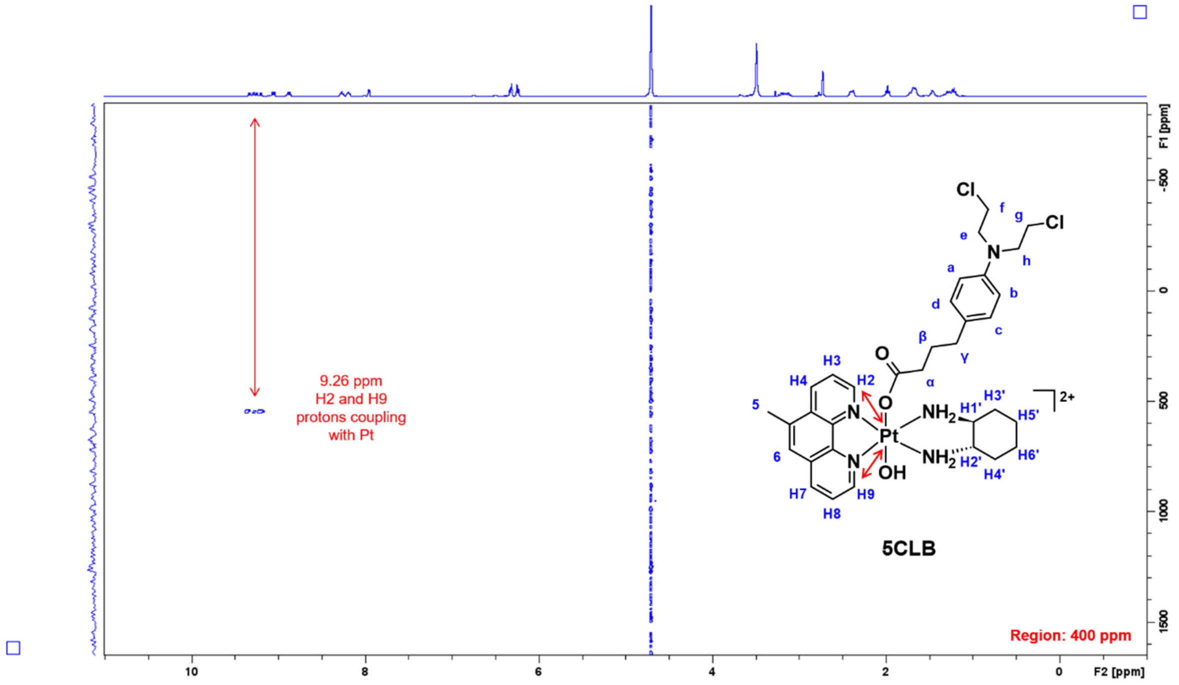

Figure S19: ^{1}H-^{195}Pt-HMQC spectrum of **5CLB** in D_2O obtained at 298 K. Region: 400 ppm. Inset: structure of **5CLB** with proton labelling system and arrow that indicate coupling.

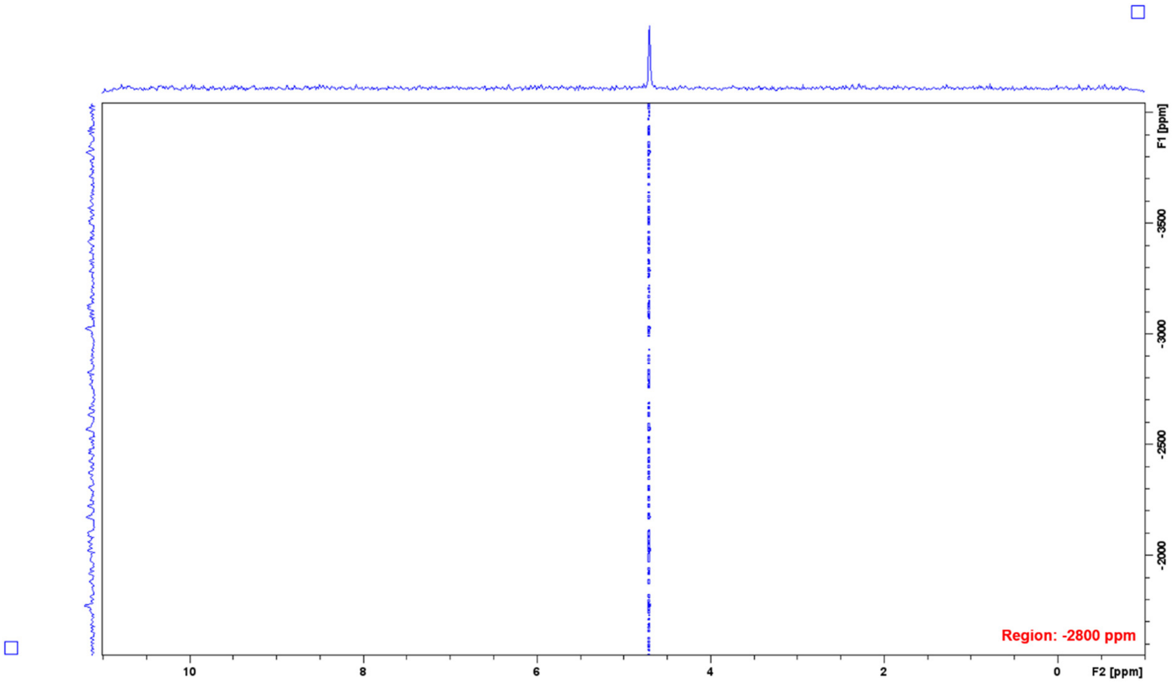

Figure S20: ^{1}H-^{195}Pt-HMQC spectrum of **5CLB** in D_2O obtained at 298 K. Region: -2800 ppm. Absence of platinum(II) resonance.

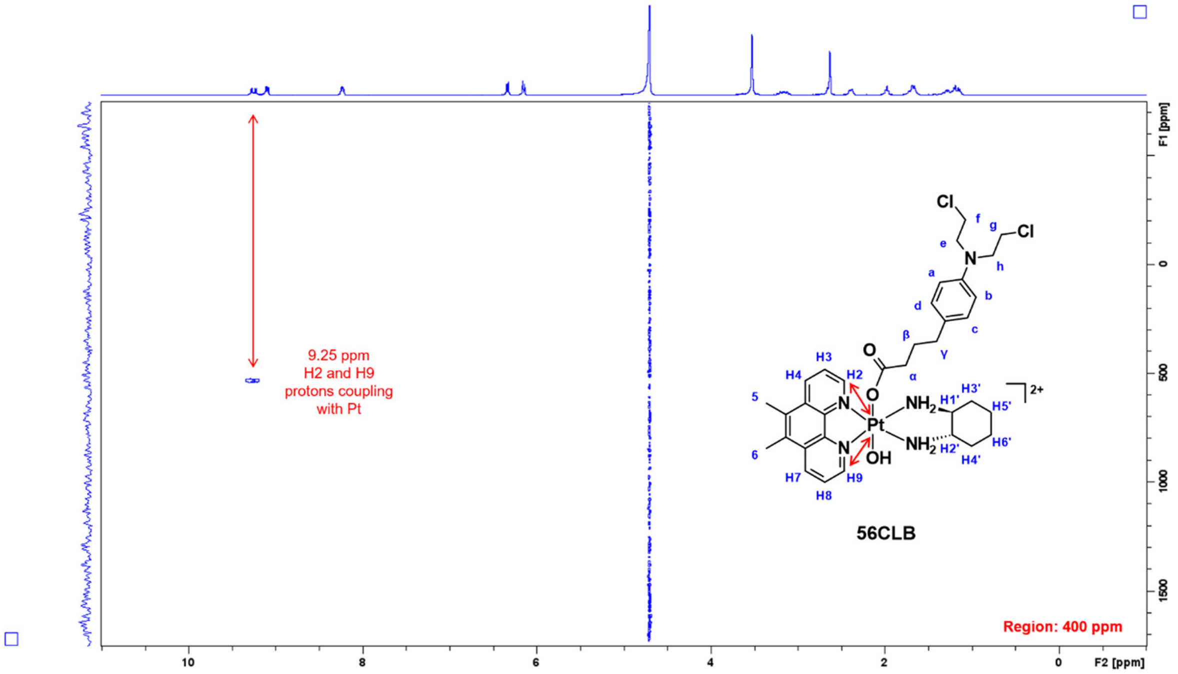

Figure S21: ^{1}H-^{195}Pt-HMQC spectrum of **56CLB** in D_2O obtained at 298 K. Region: 400 ppm. Inset: structure of **56CLB** with proton labelling system and arrows that indicate coupling.

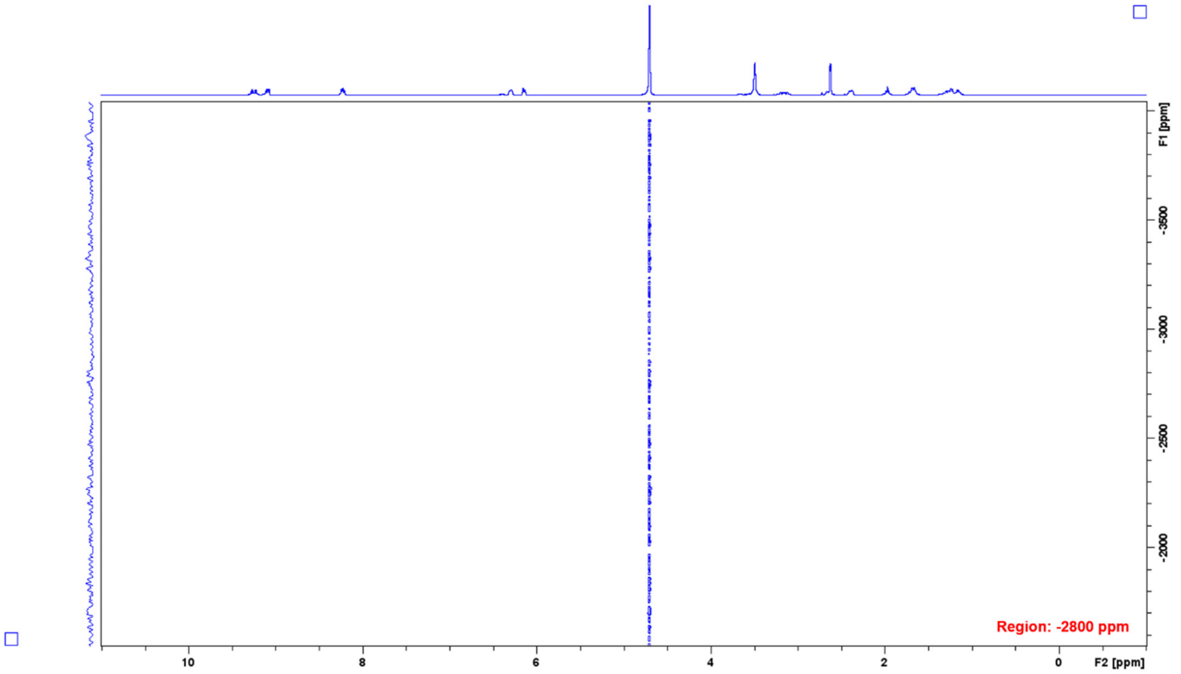

Figure S22: ^{1}H-^{195}Pt-HMQC spectrum of **56CLB** in D_2O obtained at 298 K. Region: -2800 ppm. Absence of platinum(II) resonance.

UV spectra of platinum(IV)-CLB complexes

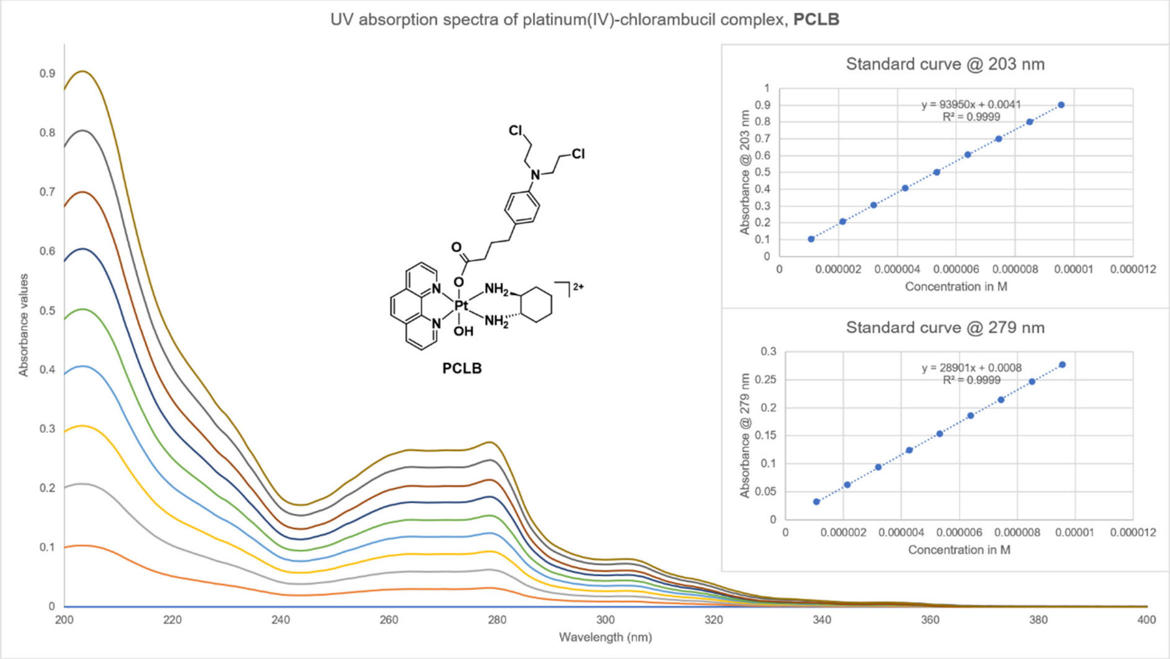

Figure S23: UV spectra of **PCLB** obtained at 298 K. Inset: structure of **PCLB** and generated plot curves within the wavelengths 203 and 279 nm at 298 K.

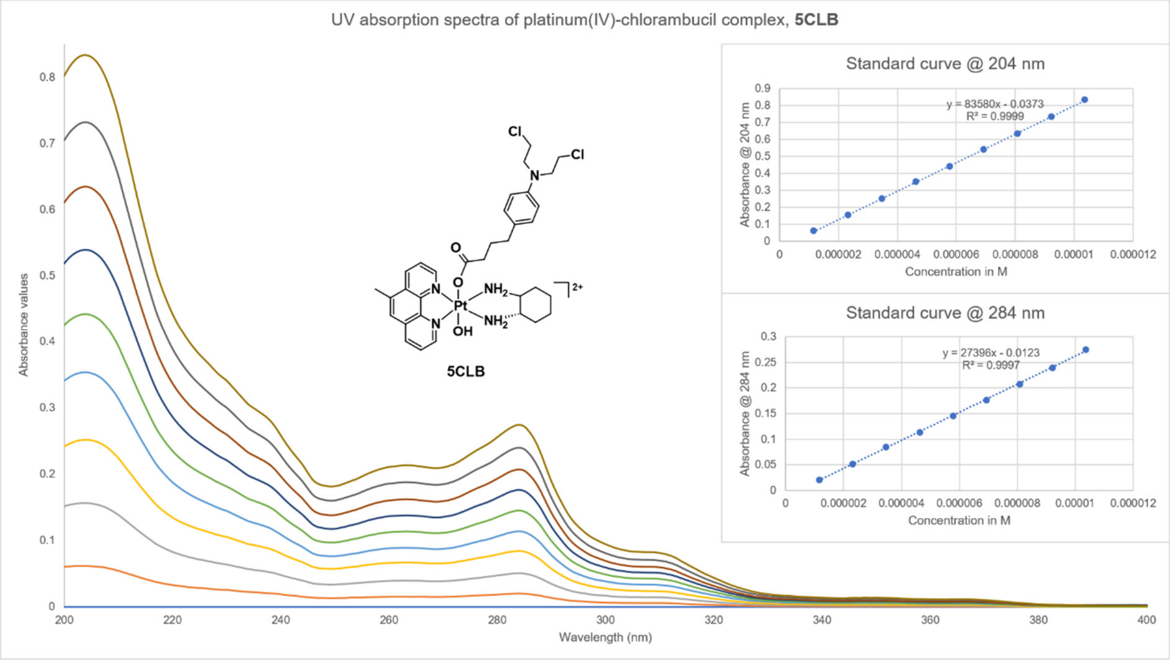

Figure S24: UV spectra of **5CLB** obtained at 298 K. Inset: structure of **5CLB** and generated plot curves within the wavelengths 204 and 284 nm at 298 K.

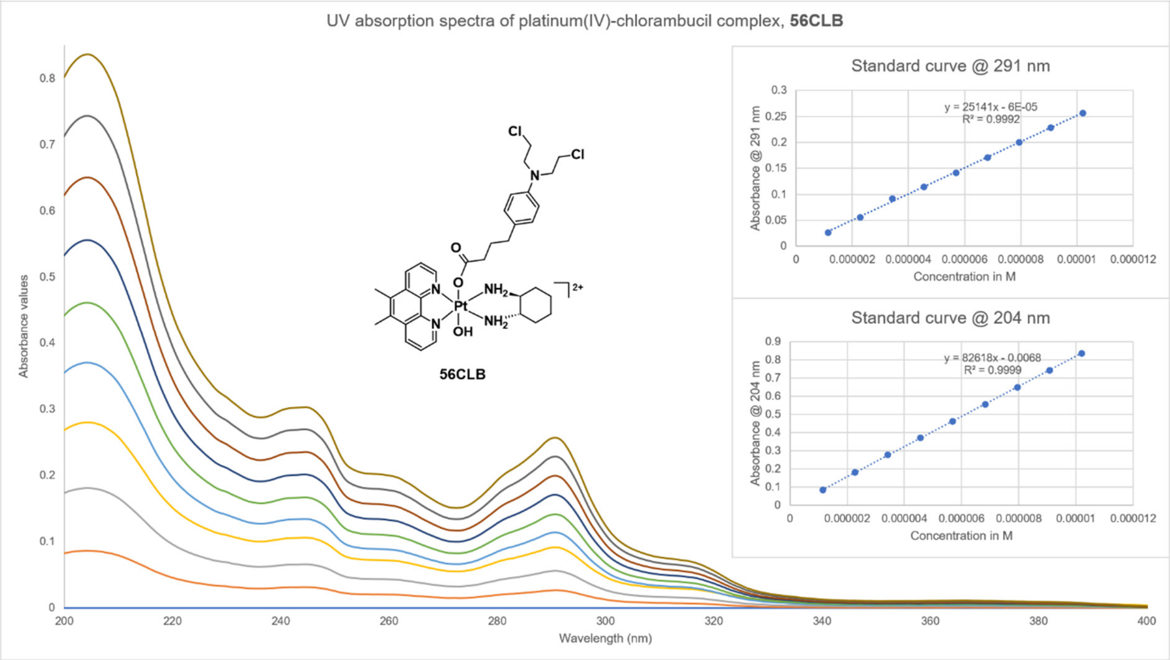

Figure S25: UV spectra of **56CLB** obtained at 298 K. Inset: structure of **56CLB** and generated plot curves within the wavelengths 204 and 291 nm at 298 K.

CD spectra of platinum(IV)-CLB complexes

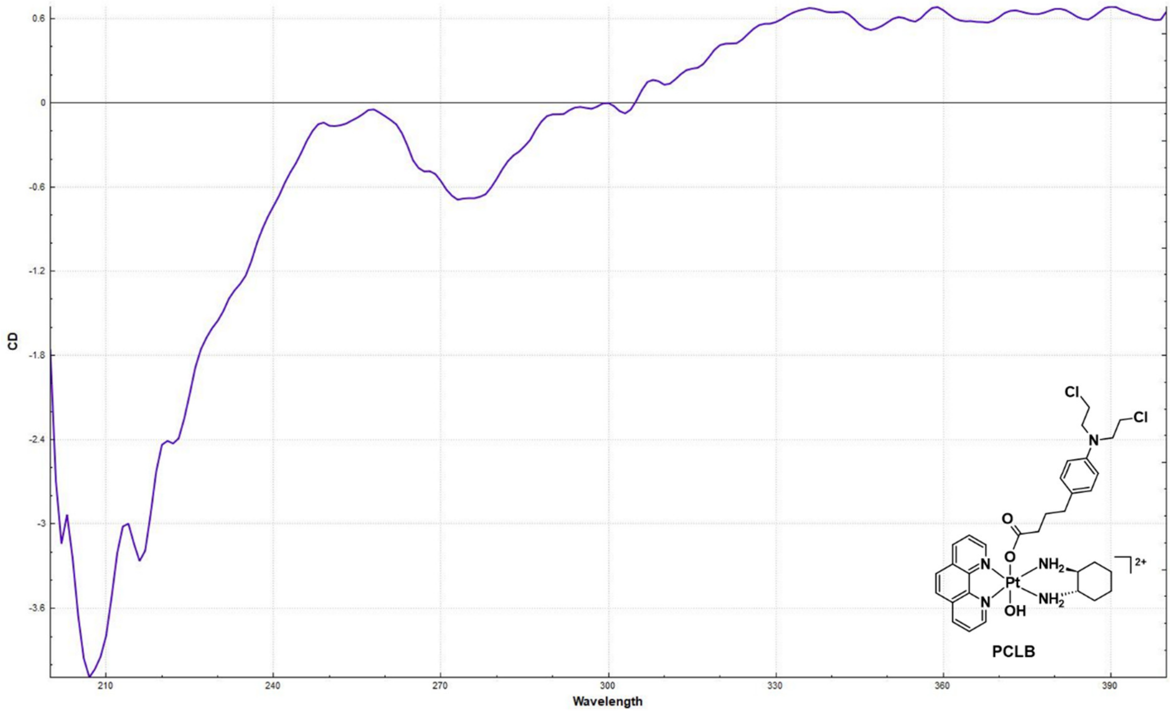

Figure S26: CD spectrum of **PCLB** in d.i.H_2O obtained at 298 K. Inset: structure of **PCLB**.

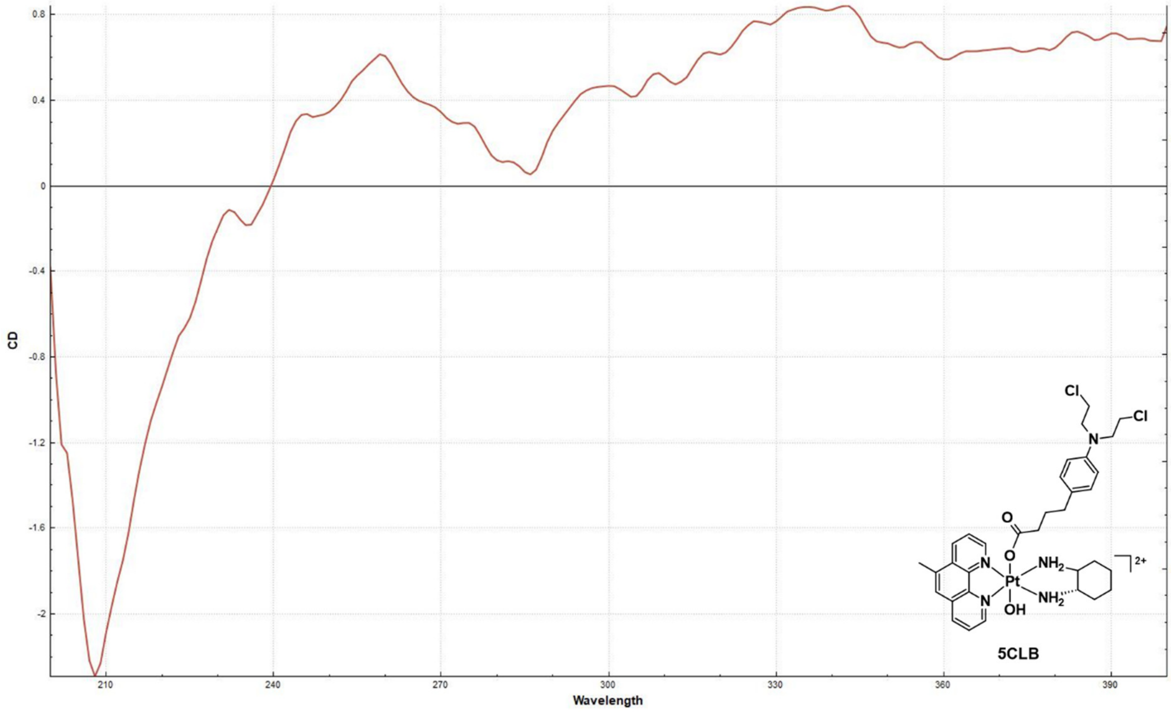

Figure S27: CD spectrum of **5CLB** in d.i.H_2O obtained at 298 K. Inset: structure of **5CLB**.

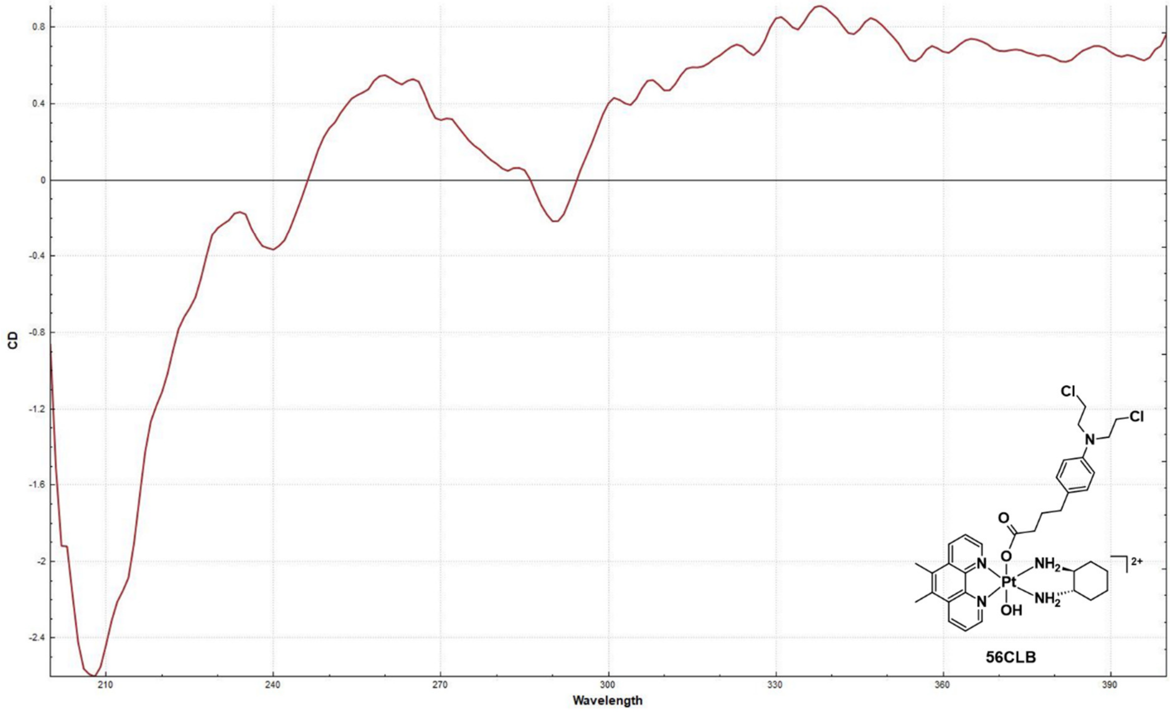

Figure S28: CD spectrum of **56CLB** in d.i.H_2O obtained at 298 K. Inset: structure of **56CLB**.

__ESI-MS spectra of platinum(IV)-CLB complexes__

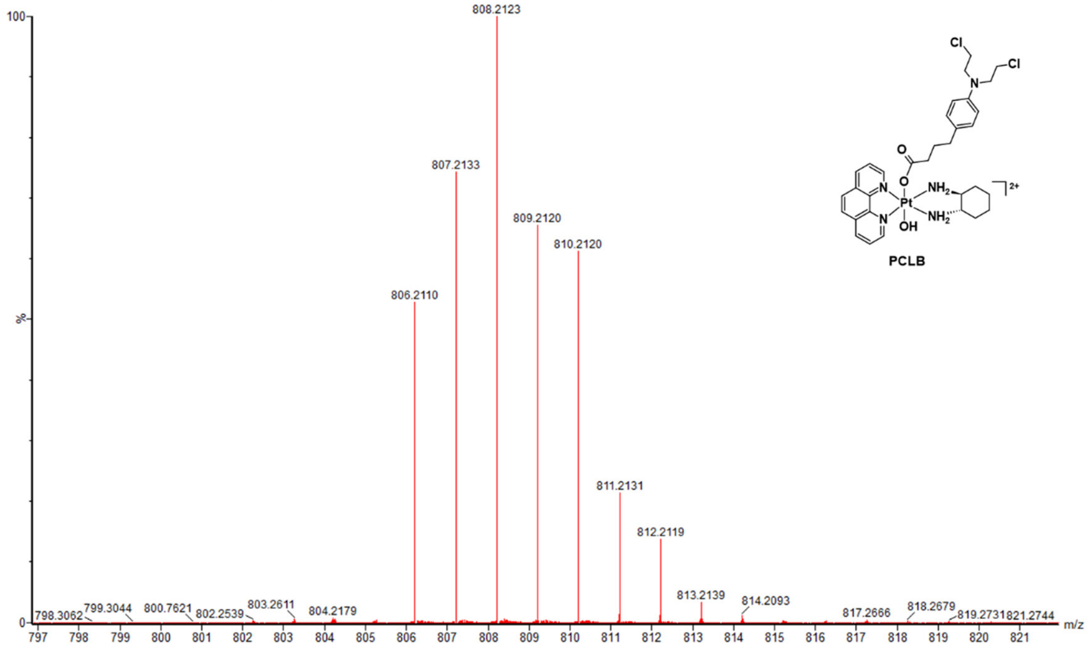

Figure S29: ESI-MS spectrum of **PCLB** in d.i.H_2O obtained at 298 K, using a Waters SYNAPT G2-Si quadruple time-of-flight (QTOF) HDMS. Inset: structure of **PCLB**.

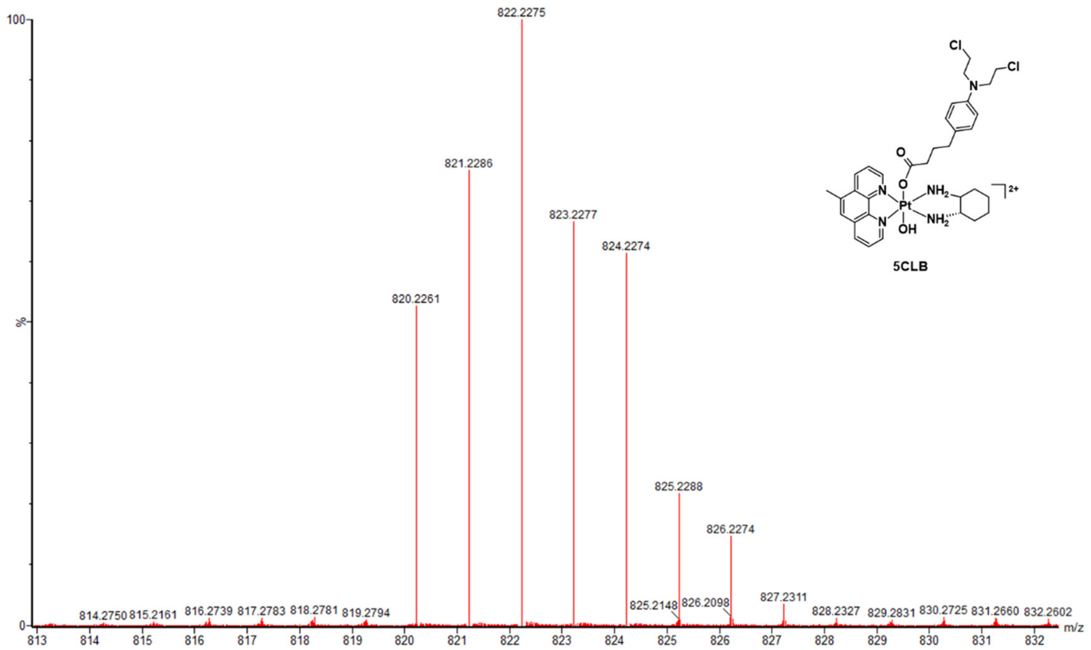

Figure S30: ESI-MS spectrum of **5CLB** in d.i.H_2O obtained at 298 K, using a Waters SYNAPT G2-Si quadruple time-of-flight (QTOF) HDMS. Inset: structure of **5CLB**.

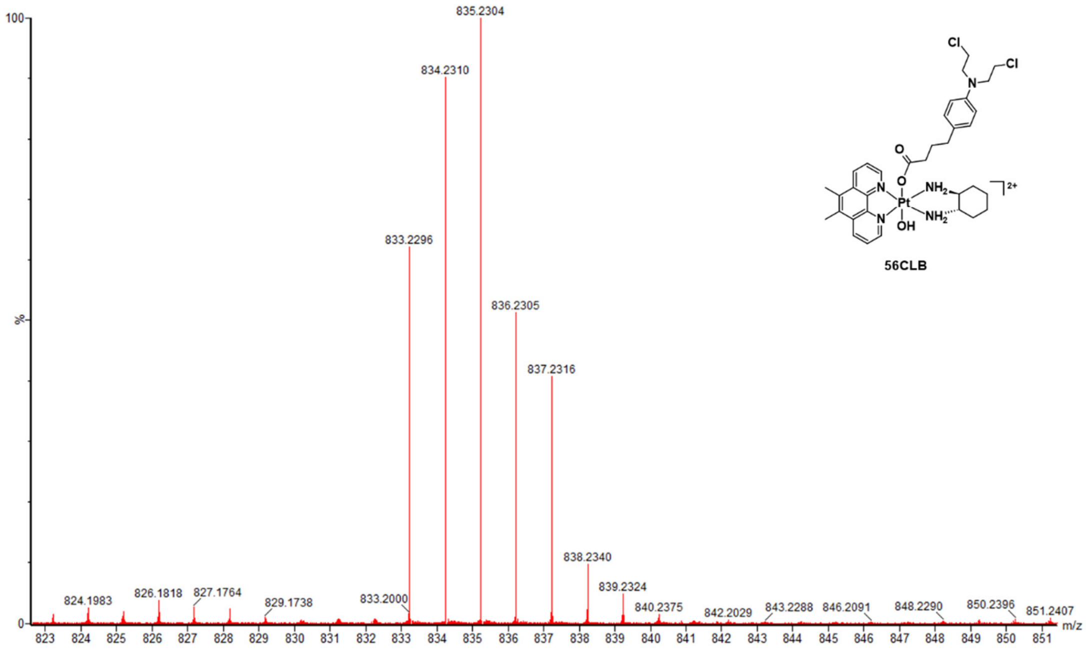

Figure S31: ESI-MS spectrum of **56CLB** in d.i.H_2O obtained at 298 K, using a Waters SYNAPT G2-Si quadruple time-of-flight (QTOF) HDMS. Inset: structure of **56CLB**.

Stability experiments

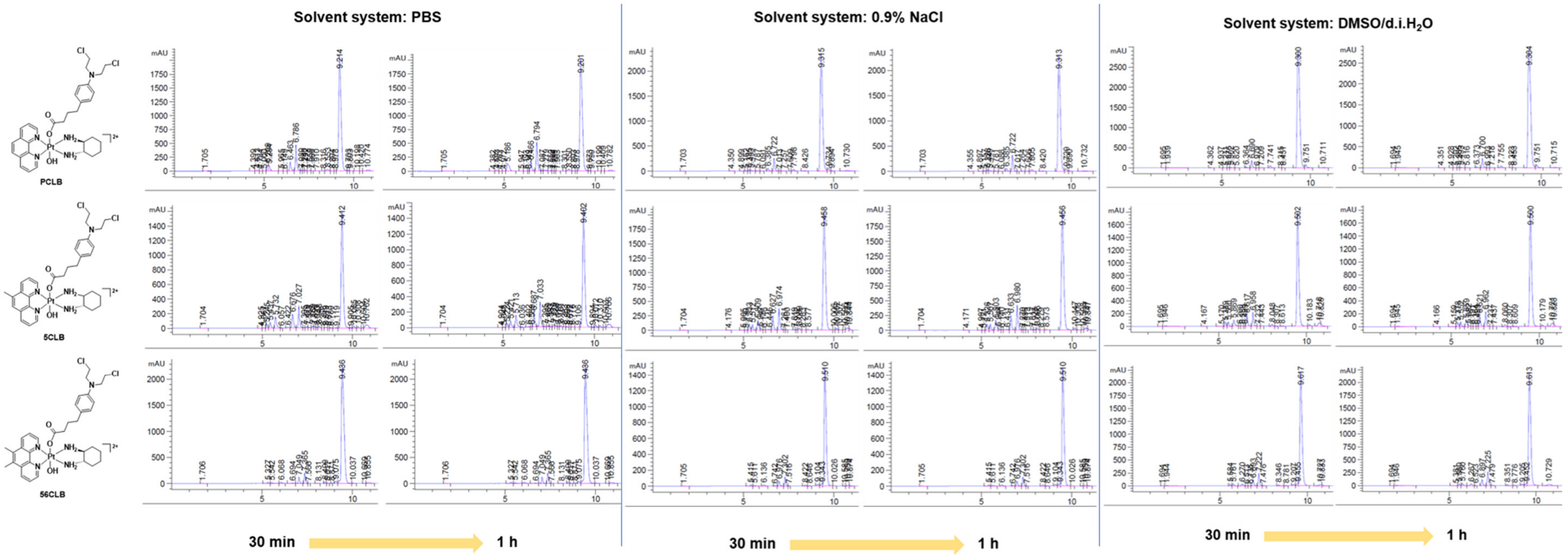

Figure S32: Combined HPLC chromatograms of **PCLB**, **5CLB** and **56CLB** in three solvent systems, obtained at 298 K. Inset: structures of **PCLB**, **5CLB** and **56CLB**.

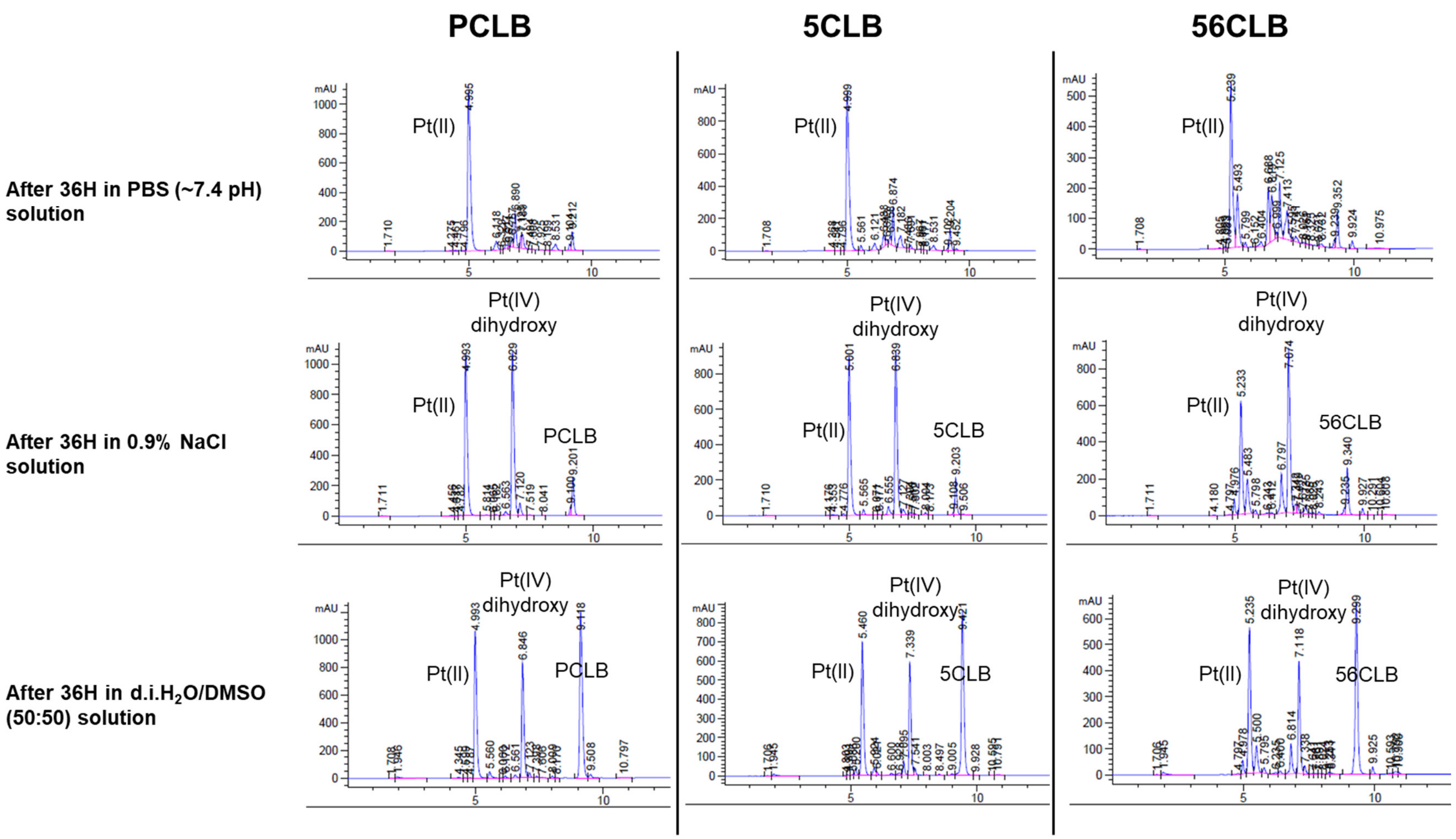

Figure S33: Combined HPLC chromatograms of **PCLB**, **5CLB** and **56CLB** in three solvent systems, obtained at 298 K at 36 h. Inset: structures of **PCLB**, **5CLB** and **56CLB**.

Lipophilicity

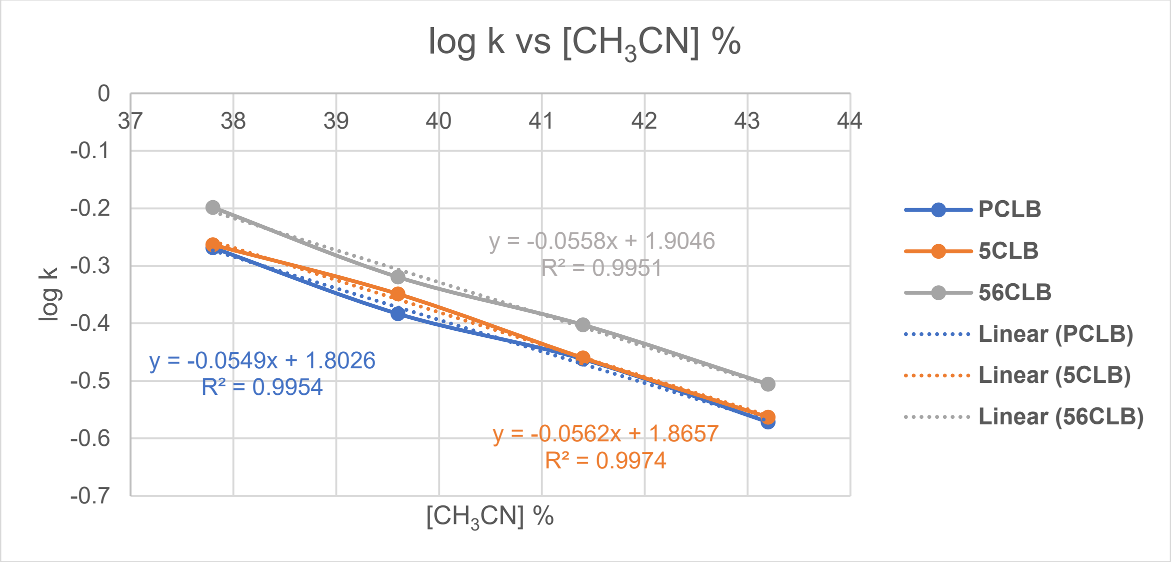

Figure S34: Generated plot curve of log k *versus* concentration of organic solvent, CH_3CN to determine the chromatographic lipophilicity index, log k_w of the platinum(IV)-CLB complexes.

__Reduction reaction experiments__

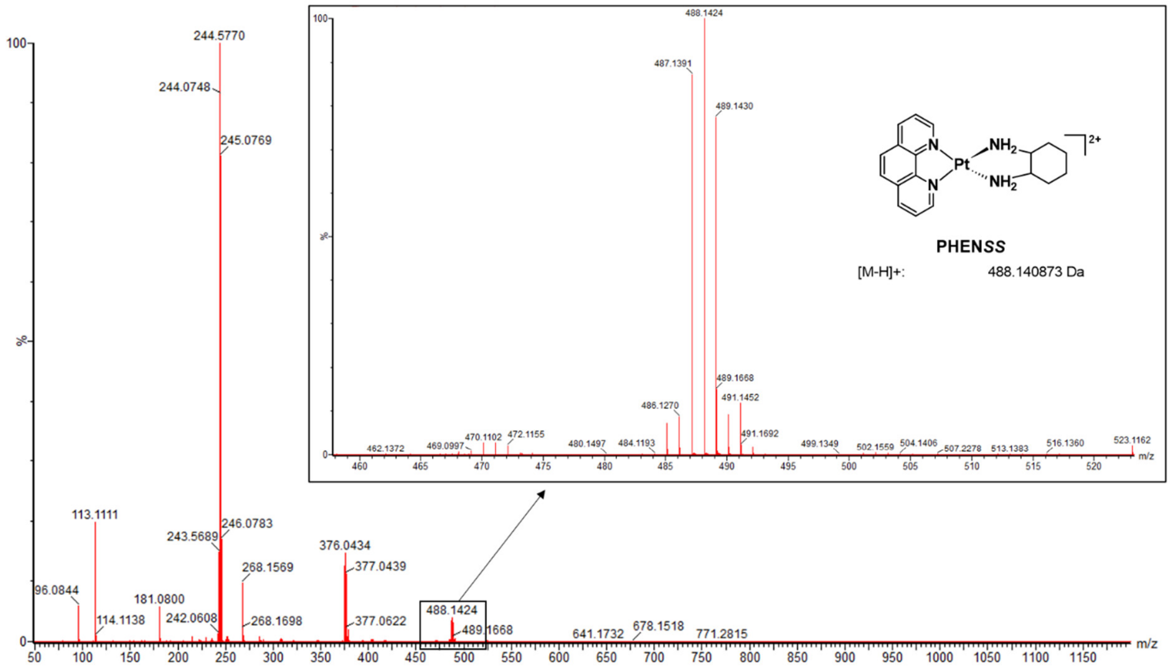

Figure S35: ESI-MS spectra of the reduced **PCLB** prodrug, highlighting the formation of the corresponding platinum(II) congener, **PHEN*SS***.

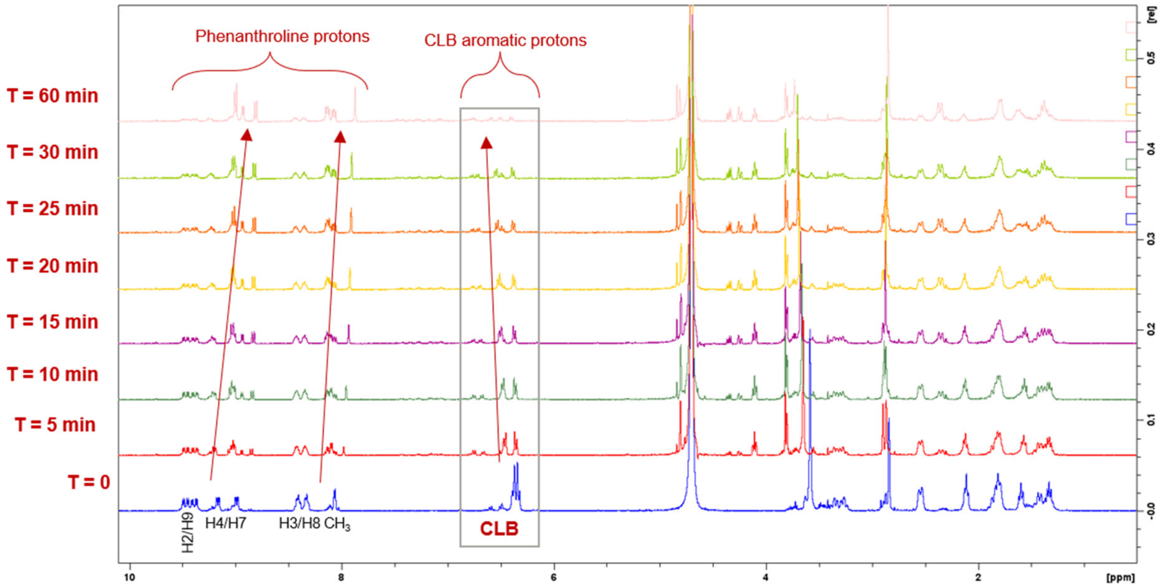

Figure S36: ^{1}H-NMR spectra of **5CLB** with PBS and AsA in D_2O at 310.15 K, in different time intervals, highlighting the movement of resonances from the phenanthroline protons and the aromatic protons of the CLB ligand. **T** represents time in min.

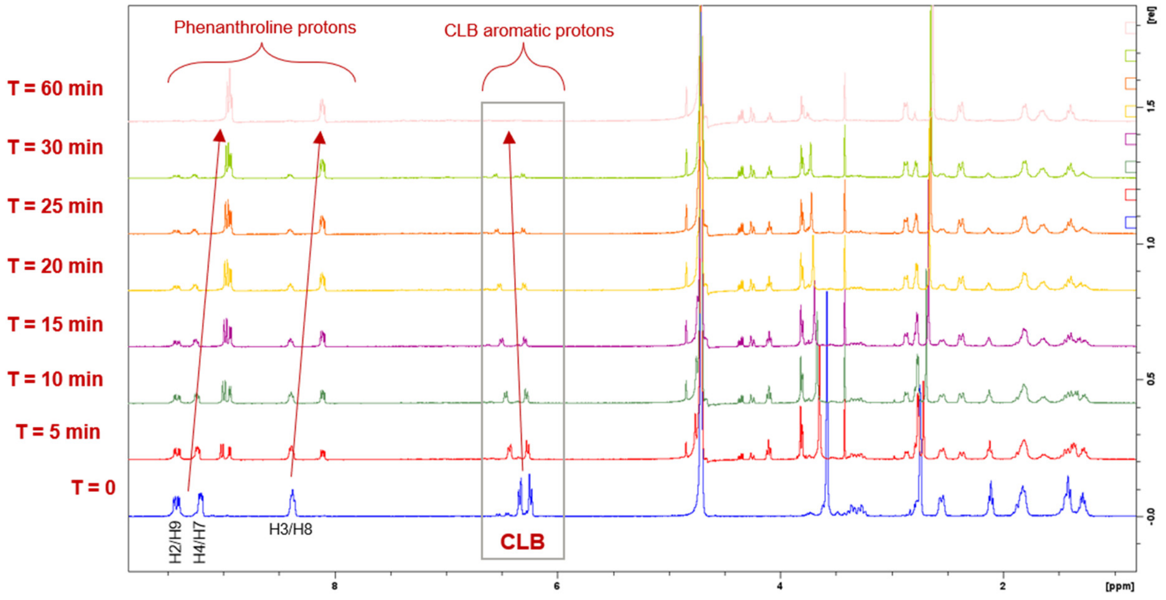

Figure S37: ^{1}H-NMR spectra of **56CLB** with PBS and AsA in D_2O at 310.15 K, in different time intervals, highlighting the movement of resonances from the phenanthroline protons and the aromatic protons of the CLB ligand. **T** represents time in min.

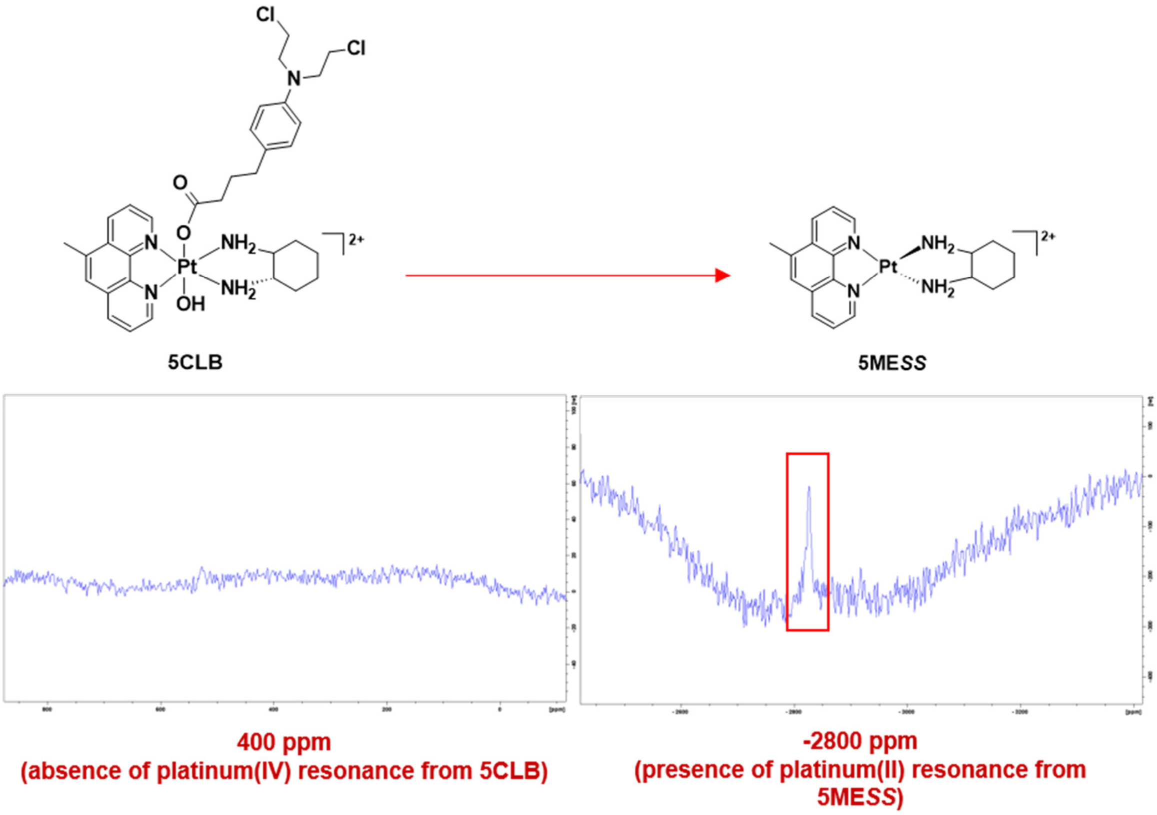

Figure S38: 1D-^{195}Pt NMR spectra of **5CLB** with PBS and AsA in D_2O at 310.15 K, within the regions of 400 and -2800 ppm, highlighting the complete reduction of the complex after 1 h. Above: Structures of **5CLB** and its platinum(II) scaffold, **5ME*SS***.

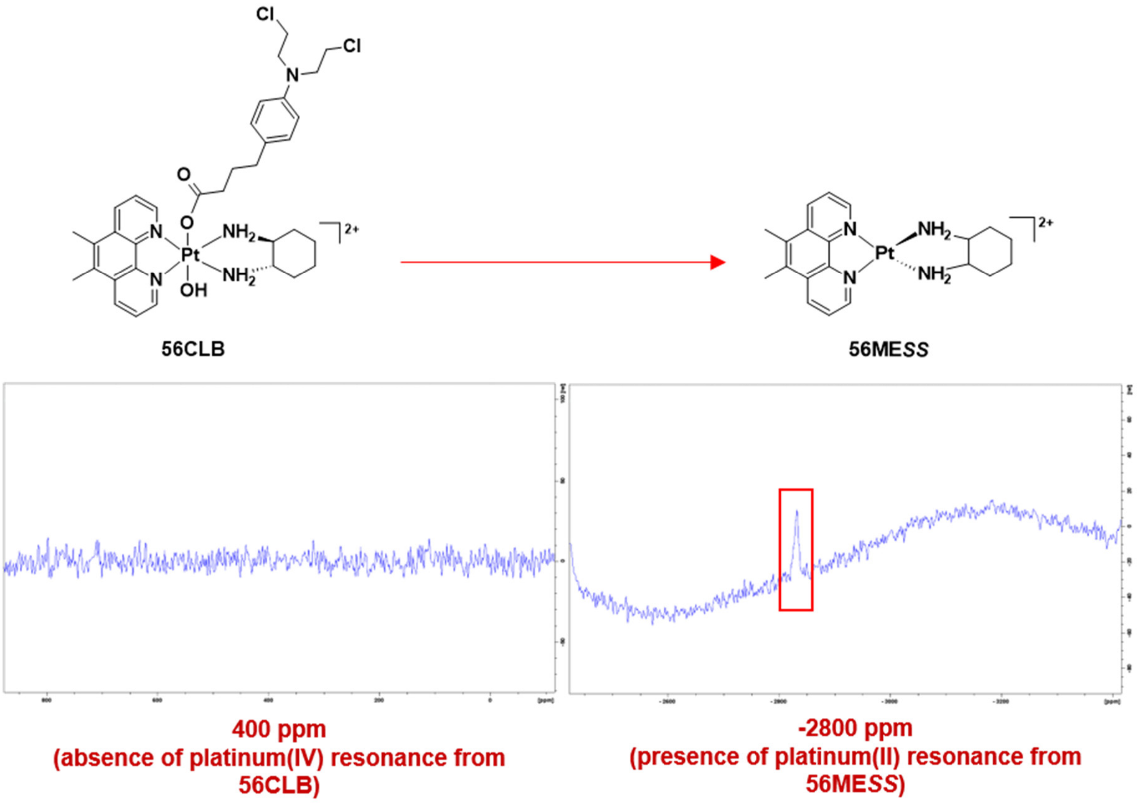

Figure S39: 1D-^{195}Pt NMR spectra of **56CLB** with PBS and AsA in D_2O at 310.15 K, within the regions of 400 and -2800 ppm, highlighting the complete reduction of the complex after 1 h. Above:Structures of **56CLB** and its platinum(II) scaffold, **56ME*SS***.

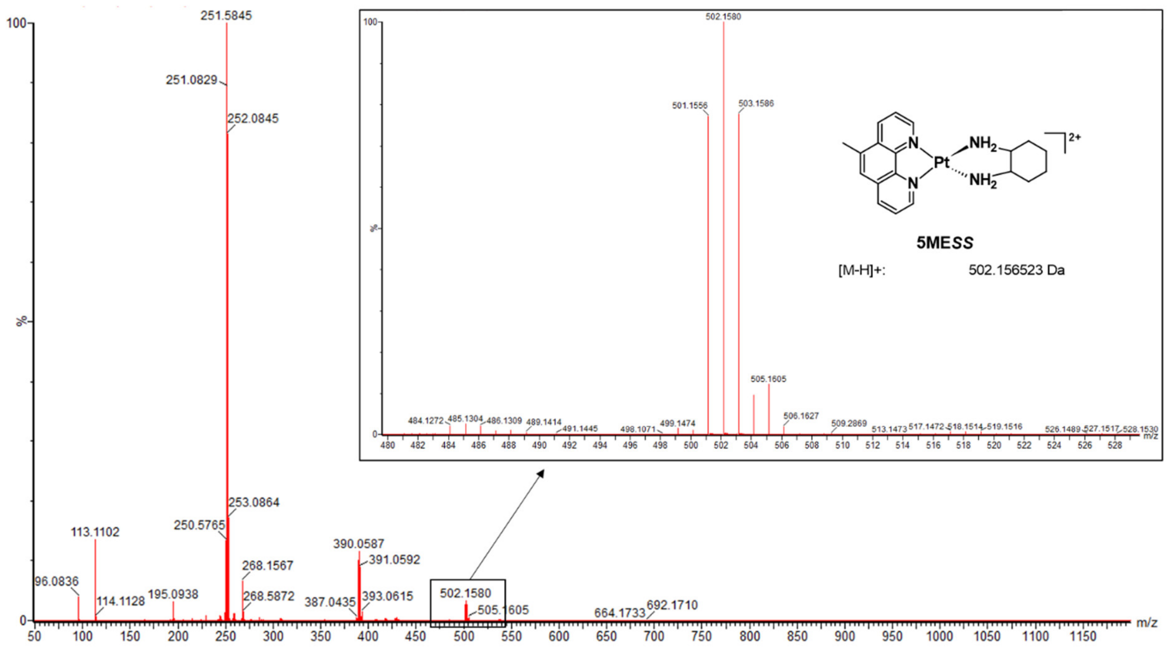

Figure S40: ESI-MS spectra of the reduced **5CLB** prodrug, highlighting the formation of the corresponding platinum(II) congener, **5ME*SS***.

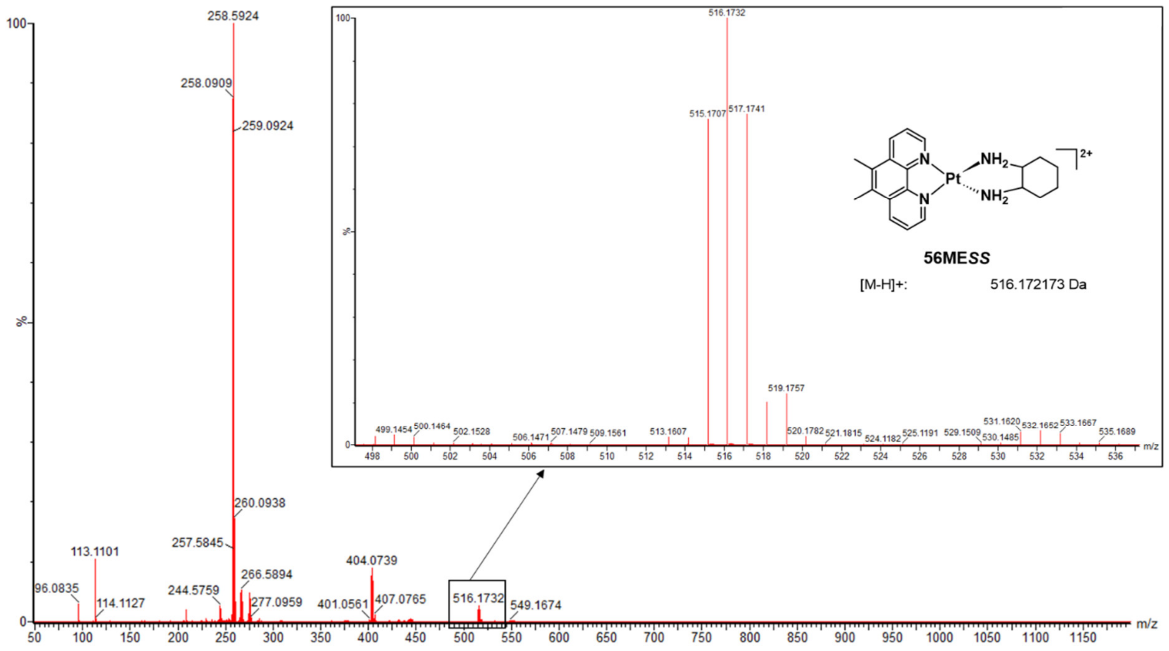

Figure S41: ESI-MS spectra of the reduced **56CLB** prodrug, highlighting the formation of the corresponding platinum(II) congener, **56ME*SS***.

ROS experiments

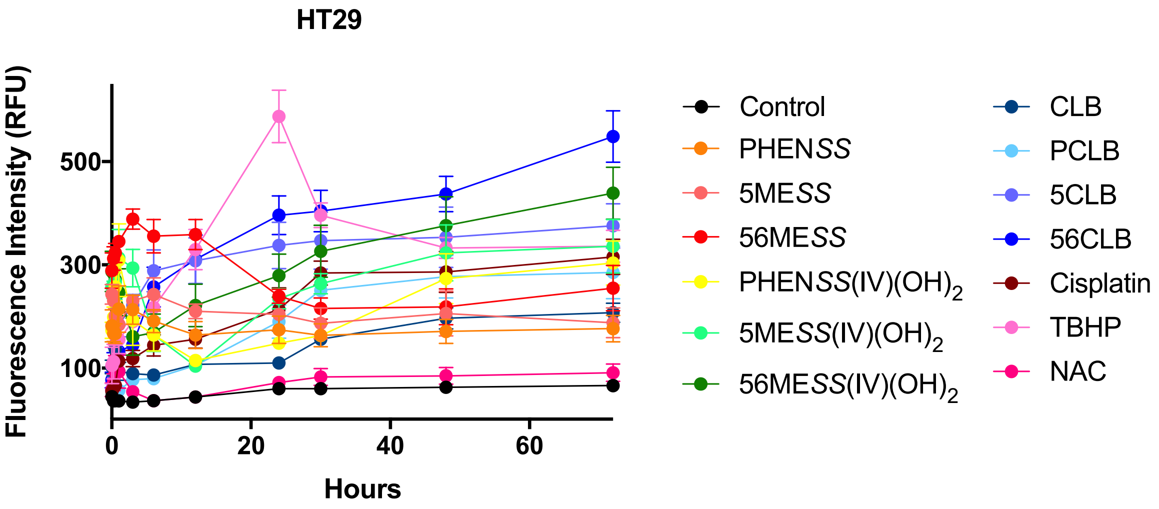

Figure S42: ROS production upon treatment with **PCLB**, **5CLB**, **56CLB**, cisplatin, together with precursor platinum(II) and (IV) scaffolds in HT29 colon cancer cell line at 0, 0.25, 0.5, 1, 3, 6, 12, 24, 48 and 72 h. TBHP: t-butyl hydroperoxide, NAC: *N*-acetylcysteine. Data points denote mean ± SEM. n = 3 from three independent experiments where samples were run in triplicates.
